# Effect of Extracellular Ribonucleic Acids on Neurovascularization in Osteoarthritis

**DOI:** 10.1002/advs.202301763

**Published:** 2023-07-03

**Authors:** Wen‐pin Qin, Qian‐Qian Wan, Jian‐Fei Yan, Xiao‐Xiao Han, Wei‐Cheng Lu, Zhang‐Yu Ma, Tao Ye, Yu‐Tao Li, Chang‐Jun Li, Chen Wang, Franklin R. Tay, Li‐Na Niu, Kai Jiao

**Affiliations:** ^1^ Department of Stomatology Tangdu hospital The Fourth Military Medical University Xi'an Shaanxi 710032 P. R. China; ^2^ State Key Laboratory of Oral & Maxillofacial Reconstruction and Regeneration & National Clinical Research Center for Oral Diseases & Shaanxi Key Laboratory of Stomatology School of Stomatology The Fourth Military Medical University Xi'an Shaanxi 710032 P. R. China; ^3^ Department of Endocrinology Endocrinology Research Center The Xiangya Hospital of Central South University Changsha Hunan 410008 P. R. China; ^4^ Department of Stomatology The Eighth Medical Center of PLA General Hospital Haidian District Beijing P. R. China 100091; ^5^ Dental College of Georgia Augusta University Augusta GA 30912 USA

**Keywords:** extracellular RNA, neurovascularization, osteoarthritis, osteochondral junction, vascular endothelial growth factor

## Abstract

Osteoarthritis is a degenerative disease characterized by abnormal neurovascularization at the osteochondral junctions, the regulatory mechanisms of which remain poorly understood. In the present study, a murine osteoarthritic model with augmented neurovascularization at the osteochondral junction is used to examine this under‐evaluated facet of degenerative joint dysfunction. Increased extracellular RNA (exRNA) content is identified in neurovascularized osteoarthritic joints. It is found that the amount of exRNA is positively correlated with the extent of neurovascularization and the expression of vascular endothelial growth factor (VEGF). In vitro binding assay and molecular docking demonstrate that synthetic RNAs bind to VEGF via electrostatic interactions. The RNA‐VEGF complex promotes the migration and function of endothelial progenitor cells and trigeminal ganglion cells. The use of VEGF and VEGFR2 inhibitors significantly inhibits the amplification of the RNA‐VEGF complex. Disruption of the RNA‐VEGF complex by RNase and polyethyleneimine reduces its in vitro activities, as well as prevents excessive neurovascularization and osteochondral deterioration in vivo. The results of the present study suggest that exRNAs may be potential targets for regulating nerve and blood vessel ingrowth under physiological and pathological joint conditions.

## Graphical abstract

During osteoarthritis progression, abnormal ingrowth of nerves and vessels occurs at the osteochondral junction. Extracellular RNA accumulates during ingrowth. Excess extracellular RNA recruits the vascular endothelial growth factor and amplifies its neurovascular stimulatory function.

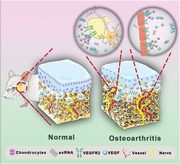

## 1 Introduction

Osteoarthritis (OA), the most common form of arthritis, affects more than 7% of the global population, corresponding to ≈500 million people worldwide. The prevalence of osteoarthritis is high in individuals of advanced age (>65 years).^[^ ^1^ ^]^ This highly debilitating condition represents a major global public health concern. Apart from large joints such as the knee and hip joints, osteoarthritis also affects small joints, such as the temporomandibular joint (TMJ). The prevalence of osteoarthritis of the TMJ is high among the elderly (≈70%), and can lead to aggravated pain and progressive dysfunction.^[^ ^2^ ^]^ Anti‐inflammatory drug therapy and surgery are the major treatment modalities for OA. However, until recently, long‐term disease remission has not been achieved, and the uncertain pathophysiological mechanisms of OA limit treatment options.

OA is a typical degenerative disease characterized by abnormal neurovascularization.^[^ ^3^ ^]^ The invasion of nerves and vessels in the osteochondral junction is one of its hallmarks and is the primary reason for aggravated pain.^[^ ^4^ ^]^ Many cytokines, including semaphorins,^[^ ^5^ ^]^ netrins,^[^ ^6^ ^]^ and growth factors such as vascular endothelial growth factor (VEGF) and nerve growth factor,^[^ ^7^, ^8^, ^9^ ^]^ have been found to play important roles in neurovascularization. Although the inhibition of a single cytokine does not completely reverse pain and OA progression,^[^ ^10^ ^]^ it is practically impossible to inhibit all cytokines involved. These issues have spurred clinical scientists to question whether other cofactors may be involved in the regulation of neurovascularization in OA.

We have previously reported that RNA‐incorporated collagen scaffolds enhance osteogenesis and angiogenesis by releasing RNA into the extracellular matrix.^[^ ^11^ ^]^ Extracellular ribonucleic acids (exRNAs) are not “cellular waste,” but indeed exert important biological functions.^[^ ^12^, ^13^ ^]^ For example, exRNAs increase vascular permeability and regeneration and promote thrombin generation and thrombus formation in cardiovascular and nervous system diseases.^[^ ^14^, ^15^, ^16^, ^17^ ^]^ Furthermore, osteoarthritic synovial fibroblasts release RNA into the extracellular matrix to inhibit the function of RNase1.^[^ ^18^, ^19^ ^]^ Further, it has been shown that intra‐articular injection of nuclear acids can induce arthritis via pannus formation.^[^ ^20^ ^]^ These findings suggest that abnormal or artificial RNA accumulation in the extracellular matrix promotes neurovascularization in OA. However, this hypothesis has not yet been verified, and the mechanisms underlying this process remain unknown.

Accordingly, the objective of this study was to test the central hypothesis that exRNAs are involved in VEGF function and neurovascular ingrowth during OA progression, for which we used a well‐established murine model of OA.^[^ ^21^, ^22^, ^23^, ^24^ ^]^ Mechanistic investigations were conducted using physicochemical characterization, molecular docking analysis, and functional verification. Additionally, exRNA scavengers were used for in vitro and in vivo investigations to test their feasibility for clinical applications through the inhibition of neurovascularization.

## 2 Results and Discussion

### 2.1 Extent of Neurovascularization in Osteoarthritic TMJs

**Figure 1:**
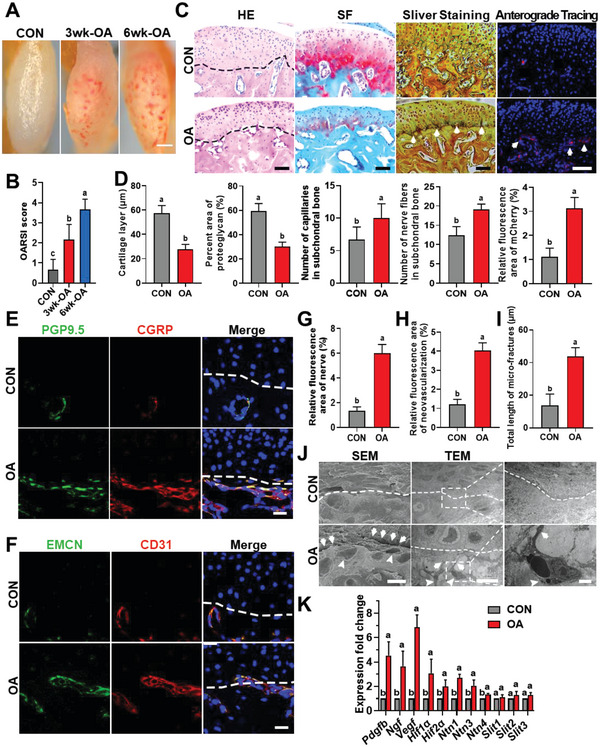
Extent of neurovascularization in OA. A,B) Representative photographs of the condyles derived from C57BL/6 mice (viewed from the top) (A), and the corresponding OARSI scores in each group (B). C,D) Representative images of H&E staining, SF staining, silver staining, and anterograde tracing of the murine condyles in the control (CON) and osteoarthritic (OA) groups at 3 weeks (C), with the corresponding statistical results (D). Arrows indicate nerve fibers. E) Representative images of PGP9.5 (green) and CGRP (red) co‐stained cells along the TMJ osteochondral junction in the CON and OA groups, at 3 weeks. F) Representative images of EMCN (green) and CD31 (red) co‐stained cells along the TMJ osteochondral junction in the CON and OA groups at 3 weeks. G,H) Semi‐statistical analysis of nerve (G) and neovascularization (H) in (E) and (F), respectively. I,J) Representative images of SEM and TEM of the mouse condyles in the CON group, and unilateral anterior crossbite (UAC) group, at 3 weeks (I), and the corresponding statistical results (J). Arrows and arrow heads in the SEM image indicate micro‐fractures and bone resorption pits, respectively. Arrows and arrow heads in the TEM image indicate new blood vessels and their accompanying nerves, respectively. K) qRT‐PCR analysis of the gene expression of neuro‐vascularization factors (*Pdgfb*, *Ngf*, *Vegf*, *Hif1α*, *Hif2α*, *Ntn1*, *Ntn3*, *Ntn4*, *Slit1*, *Slit3*, *Slit4*) in the condylar cartilage and subchondral bone in the two groups. Scale bars = 1 mm (A), 70 µm (C), 20 µm (E,F), and 10 µm (J). Data are shown as the means and standard deviations; *p* < 0.05 (*n* = 3). Different letters indicated statistically significant differences.

Osteoarthritic changes in murine TMJs were induced using a previously reported biomechanical dental stimulation method.^[^ ^21^, ^22^, ^23^, ^24^ ^]^ Condyles harvested from the 3‐week‐old osteoarthritic group (3wk‐OA), 6‐week‐old osteoarthritic group (6wk‐OA), and sham control group (CON) were used to examine the characteristics of neurovascularization. Stereomicroscopic observation identified significantly increased, randomly scattered new vessels in the 3wk‐OA and 6wk‐OA groups (*p* < 0.001). In contrast, only a few new vessels were observed in the CON group (**Figure** **1**A,B). Increased OARSI scores, increased propensity for subchondral bone capillaries, and decreased cartilage thickness and proteoglycan content were all observed in 3‐week‐old osteoarthritic condyles (all *p* < 0.05, Figure 1C,D). After confirming these osteoarthritic changes, the neurovascularization characteristics of the condyles were examined using glycine silver staining and anterograde tracing, which revealed no significant increases in nerve distribution along the osteochondral junction of the osteoarthritic joints (all *p* < 0.05, Figure 1C,D).

Sensory nerve innervation and neovascularization were observed by immunohistochemical co‐staining of calcitonin gene‐related peptide (CGRP) and protein gene product 9.5 (PGP9.5),^[^ ^25^, ^26^ ^]^ or CD31 and endomucin (EMCN).^[^ ^27^ ^]^ Compared with the CON group, the CGRP and PGP9.5 co‐stained areas (Figure 1E,G), as well as the CD31 and endomucin co‐stained areas (Figure 1F,H), were both significantly increased in osteoarthritic joints (*p* < 0.001). Significantly more microcracks, fissures, and bone resorption pits were identified with scanning electron microscopy in the OA group (*p* < 0.001, Figure 1I,J). Transmission electron microscopy revealed the infiltration of the pseudopodia of epithelial cells and neurons into the osteochondral junction of the osteoarthritic joints (Figure 1J). Collectively, these results indicate that increased neurovascularization originated from the subchondral bone. These new neurovascular buds breach the tidemark and accelerate cartilage collapse and progression of OA.^[^ ^25^, ^26^, ^27^, ^28^, ^29^, ^30^, ^31^ ^]^

Quantitative reverse transcription polymerase chain reaction (qRT‐PCR) was performed to examine the expression of neurovascular cytokines in the condyles of the CON and OA groups. Various growth factors and axon guidance factors were found to be upregulated in the osteoarthritic condyles (*p* < 0.001, Figure 1K), thereby confirming that the condylar microenvironment is pro‐angiogenic and pro‐neurogenic during OA progression. Among the neurovascular cytokines, VEGF expression significantly increased (*p* < 0.001). Because VEGF facilitates the ingrowth of blood vessels and promotes neurogenesis,^[^ ^32^ ^]^ while arteriogenesis is co‐dependent on VEGF and exRNA,^[^ ^14^ ^]^ it was speculated that exRNA promotes neurovascularization in OA.

### 2.2 Relationship between exRNA Levels and Neurovascularization in Osteoarthritic Joints

**Figure 2:**
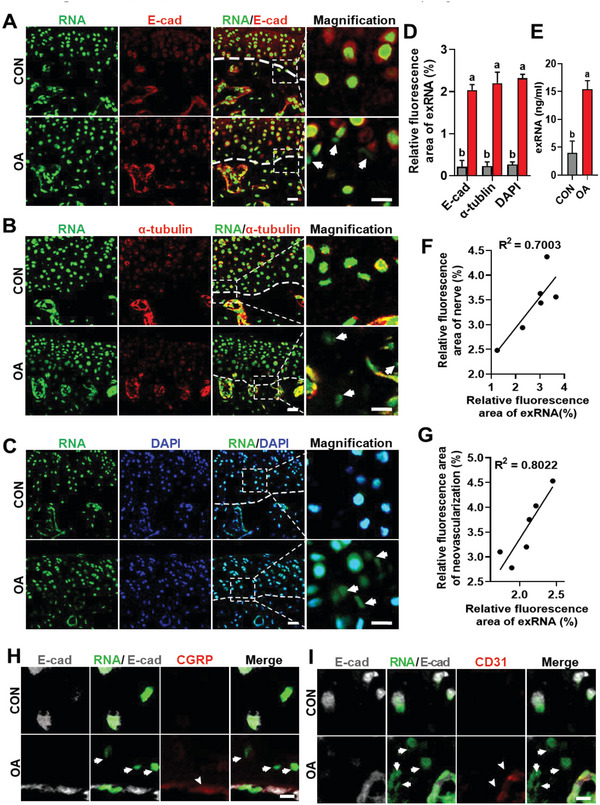
Neuro‐vascularization was accompanied by a local increase in exRNA. A–C) Representative CLSM images of the distribution of exRNA along the osteochondral junction of TMJs derived from the CON and OA groups at 3 weeks. RNA, green; E‐cadherin and *α*‐tublin, red; DAPI, blue. Arrows denote exRNA. D) Semi‐statistical analysis of exRNA (C,D) results. E) Statistical analysis of exRNA in the condylar cartilage and subchondral bone of the two groups at 3 weeks. F) Pearson correlation analysis of the relative fluorescent areas occupied by nerves and exRNA (*n* = 6, *R* ^2^ = 0.7003, *p* = 0.0377). Similar analysis of the relative fluorescent areas occupied by neovascularization and exRNA in G (*n* = 6, *R* ^2^ = 0.8022, *p* = 0.0158). H,I) CLSM images taken from the osteochondral junction showing that exRNA is adjacent to the nerves (H) and blood vessels (I). Arrows indicated exRNA and arrowheads represented nerves and vessels. Scale bars = 20 µm (A–C) and 15 µm (H,I). Data are shown as the mean and standard deviation; *p* < 0.05 (*n* = 3). Different letters indicated statistically significant differences.

The release of exRNAs from synovial fibroblasts occurs in various types of arthritis,^[^ ^18^ ^]^ and the origin of these exRNAs may be related to cell death.^[^ ^33^ ^]^ In addtion, vital cells release exRNA when stimulated by damage‐related signals.^[^ ^14^, ^18^ ^]^ To identify exRNA in the osteochondral junction of the osteoarthritic TMJs, sections of the condyles from the 3wk‐OA group were co‐stained with SYTO RNASelect Green Fluorescent Cell Stain (an RNA‐specific stain), E‐cadherin (cytomembrane), *α*‐tubulin (cytoskeleton), or 4',6‐Diamidino‐2'‐phenylindole (DAPI, nucleus). In the OA group, exRNA was distributed in the vicinity of the osteochondral junction in areas distant from those stained for E‐cadherin (**Figure** **2**A), *α*‐tubulin (Figure 2B), and DAPI (Figure 2C). These features were negligible in the CON group (*p* < 0.001; Figure 2D). The exRNA from the cells within the osteochondral junction of the two groups was isolated using magnetic beads without rupturing the cell membranes, showing a significant increase in the exRNA content in the osteoarthritic group (*p* < 0.001, Figure 2E).

Unlike the condyles derived from the CON group, the amount and activity of RNase in the osteoarthritic condyles were significantly reduced (*p* < 0.05; Figure S1, Supporting Information). Pearson's correlation analysis revealed a significant positive correlation between areas displaying exRNA‐labeled fluorescence and areas occupied by CGRP^+^ and PGP9.5^+^ sensory nerves (*R* ^2^ = 0.700, *p* < 0.05, Figure 2F). Similarly, a significant positive correlation was detected between the areas displaying exRNA‐labeled fluorescence and those occupied by CD31^+^ and EMCN^+^ H‐type vessels (*R* ^2^ = 0.802, *p* < 0.05, Figure 2G). Confocal laser scanning microscopy (CLSM) of the co‐stained images also revealed close relationships between the locations of exRNA and nerves and between the location of exRNA and blood vessels (Figure 2H,I; Figures S2 and S3, Supporting Information). Taken together, these results indicate that the spatial distribution and amount of exRNA are positively correlated with neurovascularization.

### 2.3 Mechanism Whereby ExRNA Engaged in Neurovascularization

**Figure 3:**
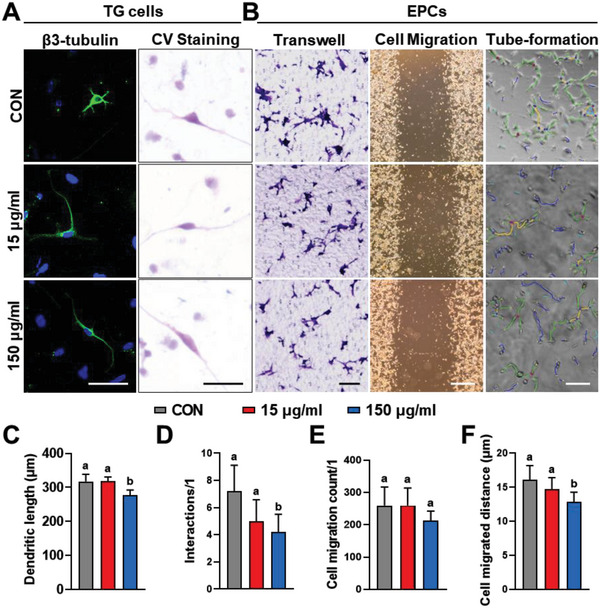
exRNA does not directly promote the function of TG cells and EPCs. A) Representative microscopy images of TG cells showing the length of their dendrites and interactions after treatment for 24 h. B) Representative light microscopy images of EPCs showing migrated cells, cell migration, and tube formation, after treatment for 24 h. C,D) Quantification of dendritic length and interactions of TGs. E,F) Quantification of the numbers and distance of migrated EPCs. Scale bars = 50 µm (A), 100 µm (left and right in B), and 500 µm (middle in B). Data are shown as the mean and standard deviation; *p* < 0.05 (*n* = 3). Different letters indicate statistically significant differences.

In vitro experiments were conducted to examine the effects of exRNAs on neurogenesis and angiogenesis. Most exRNAs were less than 60 nucleotide units (nt) in length.^[^ ^34^ ^]^ The in vivo concentrations of the exRNAs were also minimal (<100 ng mL^−1^).^[^ ^14^ ^]^ Hence, endothelial progenitor cells (EPCs) and trigeminal ganglion primary cells (TGs) were treated with a low (15 ng mL^−1^) or high (150 ng mL^−1^) concentration of synthetic RNA (50 nt) for 24 h in vitro. Low RNA concentrations did not affect the functions of EPCs and TGs; however, high RNA concentrations exerted inhibitory effects on both cell types (all *p* < 0.05; **Figure** **3**). Because a previous in vivo experiment demonstrated a positive correlation between exRNA content and neurovascularization, we speculated that exRNAs did not function directly in neurovascularization.

Ribonucleic acid–protein complexes play important roles in many cellular processes, including protein synthesis and gene regulation.^[^ ^35^, ^36^ ^]^ For example, nucleic acids cause the phase separation of positively charged proteins.^[^ ^37^ ^]^ These proteins, in turn, stabilize nucleic acids and protect them from degradation. Ribonucleic acids direct certain proteins to suitable subcellular positions to regulate specific downstream protein–protein interactions.^[^ ^38^ ^]^

**Figure 4:**
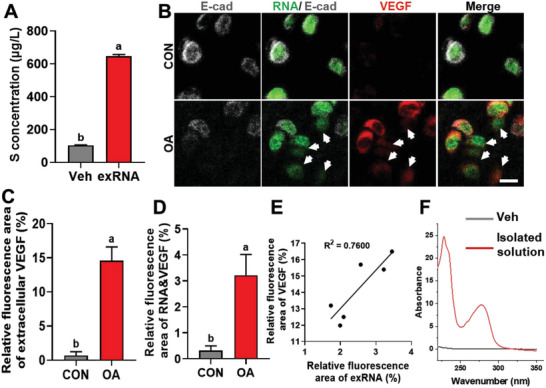
exRNA co‐localized with VEGF at the osteochondral junction. A) exRNA isolated from osteoarthritic condyles were examined with ICP‐MS to determine the concentration of the sulfur element. Veh refers to the vehicle (i.e., the elution buffer). B) Representative images illustrating the co‐localization of exRNA and VEGF at the osteochondral junction of osteoarthritic TMJs. Arrows indicated the co‐localization of exRNA and VEGF. C,D) The relative fluorescence area of VEGF (C) and the co‐localization of exRNA and VEGF (D) results. E) Pearson correlation analysis of the relative fluorescence area of VEGF and exRNA (*n* = 6, *R* ^2^ = 0.7600, *p* = 0.0236). F) The NanoDrop test verified the existence of the OD260‐positive fraction in the eluate containing the VEGF‐antibody affinity purified complex. Veh referred to vehicle (i.e., the 0.1 m glycine solution). Scale bars = 15 µm (B). Data are shown as the means and standard deviations; *p* < 0.05 (*n* = 3). Different letters indicate statistically significant differences.

To examine whether exRNA participates in neurovascularization through RNA‐protein interactions, exRNA was harvested from the local microenvironment of osteoarthritic condyles. The elemental content of the harvested exRNAs was analyzed using inductively coupled plasma‐mass spectrometry. As illustrated in **Figure** **4**A, sulfur was detected in the exRNA solution derived from osteoarthritic condyles (*p* < 0.001). Because sulfur was absent in the nucleotides and was present only in extracellular proteins with abundant disulfide bridges,^[^ ^39^ ^]^ it is possible that the exRNAs interacted with extracellular proteins to promote neurovascularization. The GSE45793 dataset was subsequently analyzed to identify potential neurovascular proteins that interact with the exRNAs.^[^ ^40^ ^]^ Based on the fold‐change and *p* value, VEGF was identified as the most likely protein candidate (Table S1, Supporting Information). This result is consistent with the corresponding qRT‐PCR results (Figure 1K).

Immunofluorescence staining and biochemical experiments were performed in vivo to determine the relationship between the exRNAs and VEGF. An increase in extracellular VEGF expression, as well as the co‐localization of exRNA and VEGF, was clearly observed (white arrow) (all *p* < 0.001, Figure 4B–D; Figure S4, Supporting Information). Pearson's correlation analysis also revealed a significant positive correlation between the relative fluorescence area of exRNA and VEGF in serial sections (*R* ^2^ = 0.760, *p* < 0.05, Figure 4E).

VEGF was isolated from osteoarthritic condyles using biotin‐labeled VEGF antibodies and streptavidin‐coupled magnetic beads. After elution, eluate containing the VEGF‐antibody affinity purified complex (designated as the “VEGF solution” group), and the pure eluate (0.1 m glycine, designated as the “Veh” group) were examined by western blot and NanoDrop test. An OD260 peak was detected for the VEGF solution (Figure 4F; Figure S5, Supporting Information). As the VEGF protein only possessed an OD280 peak, the existence of an OD260‐positive fraction was consistent with the presence of nucleic acids. Taken together, these results suggest a potential interaction between exRNAs and VEGF in the neurovascularization of osteoarthritic condyles.

### 2.4 Interaction between ExRNA and VEGF Molecules In Vitro

**Figure 5:**
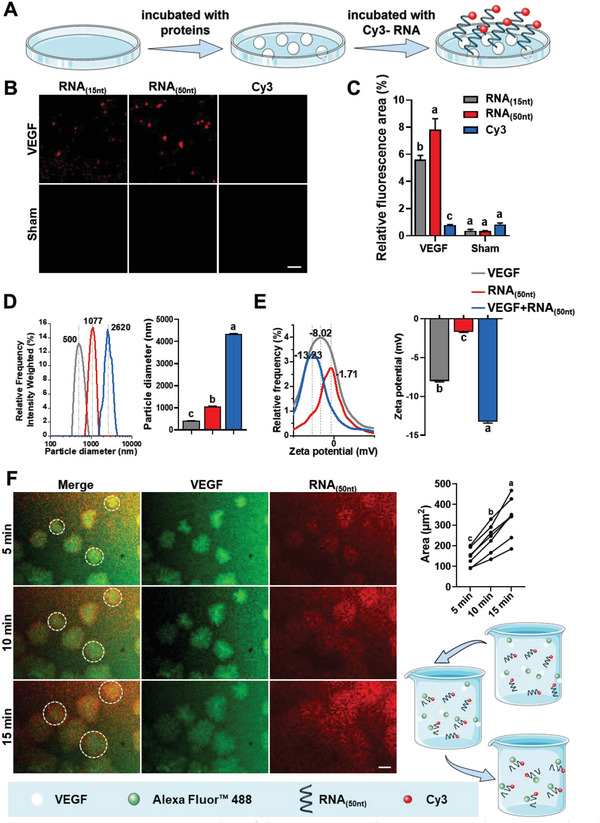
VEGF binds to RNA. A) Design of the in vitro experiment. B) CLSM images showing that VEGF binds more efficiently to RNA_(50 nt)_ than RNA_(15 nt)_. C) Data in (B) were analyzed quantitatively. D) Size distribution of VEGF, RNA_(50 nt)_, RNA_(50 nt)_‐VEGF, and the corresponding statistical analysis. E) Zeta potential of VEGF, RNA_(50 nt)_, RNA_(50 nt)_‐VEGF, and the corresponding statistical analysis. F) Time‐lapse images of the RNA_(50 nt)_‐VEGF phase separation, and the corresponding statistical results. Liquid droplets formed after mixing of recombinant VEGF (Alexa Fluor 488‐labeled) with RNA_(50 nt)_ (Cy3‐labeled) and matured over 15 min. The images shown are representative of all fields in the well. Scale bars = 30 µm (B), 10 µm (F). Data are shown as the means and standard deviations; *p* < 0.05 (*n* = 3). Different letters indicate statistically significant differences.

An in vitro model was used to validate the interaction between the exRNAs and VEGF during neurovascularization. The orange‐fluorescent dye Cy3 labeled RNA (Cy3‐RNA) was added to VGEF‐coated confocal dishes (**Figure** **5**A). The rationale for this experiment was that fluorescence would be detected only when Cy3‐RNAs were bound to VEGF molecules. Because exRNAs shorter than 60 nt consist of short (12–15 nt) and long fragments (50‐60 nt)^[^ ^34^ ^]^ Cy3‐RNAs_(15 nt)_ and Cy3‐RNAs_(50 nt)_ were used as representative for the experiment. CLSM images showed that the fluorescence intensity was stronger in the Cy3‐RNA_(50 nt)_‐VEGF group, with a statistically significant difference (*p* < 0.01, Figure 5B,C). The absence of fluorescence in the sham group excluded the possibility of direct binding between the dishes and the RNA. Similarly, the absence of fluorescence in the Cy3 group excluded the possibility of direct binding between Cy3 and VEGF (Figure 5B,C). These results indicate that the ability of RNA to recruit VEGF depends on the RNA length.

Measurements of the size distribution and zeta potential also supported the hypothesis that the RNA_(50 nt)_ effectively bound to VEGF (Figure 5D,E). In a subsequent experiment, fluorescently labelled VEGF was added to Cy3‐RNA_(50 nt)_ in solution to monitor its direct interaction. Upon mixing, VEGF and RNA formed macroscopic aggregates (mean diameter: 12 µm) within 5 min, which reached their maximum diameter (≈20 µm) within 15 min (Figure 5F). These data suggested that RNA interacts with VEGF to form aggregates in vitro. This function depends on the length of the RNA. In addition, the formation of RNA–VEGF complexes increased the stability of RNA in solution by preventing the RNAs from degradation (Figure S6, Supporting Information).

**Figure 6:**
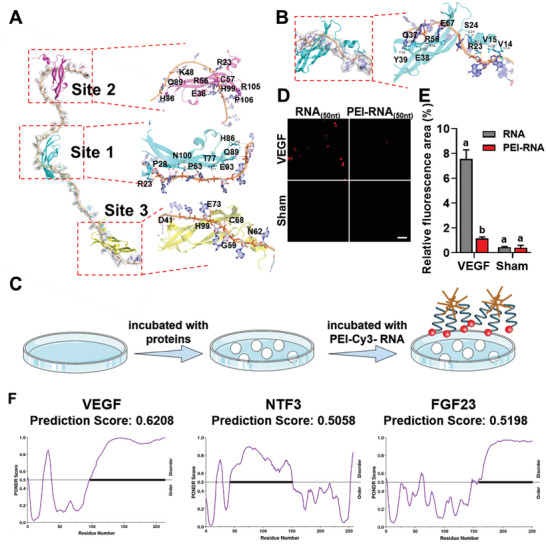
VEGF binds to RNA through electrostatic interaction. A,B) Binding patterns between RNA_(50 nt)_ and VEGF (A), and between VEGF and RNA_(15 nt)_ (B). The stick model represents RNA_(50 nt)_. The cartoon represents VEGF. Light blue represents Site 1, carmine represents Site 2, and yellow represents Site 3. C) Design of the in vitro experiment. D) CLSM images showing that the cationic polymer PEI disrupts the interaction between RNA_(50 nt)_ and VEGF. E) Data in (D) were analyzed quantitatively. Scale bars = 30 µm (D). F) Predicted intrinsically disordered regions of VEGF, NTF3, and FGF23. These processes were conducted by VSL2 in PONDR. Data are shown as the means and standard deviations; *p* < 0.05 (*n* = 3). Different letters indicate statistically significant differences.

Molecular docking was performed to identify the mode of interaction between VEGF and RNA. The structures of RNA_(15 nt)_ and RNA_(50 nt)_ were obtained from their sequences. The structure of VEGF was downloaded from the Protein Data Bank (PDB ID:1VPF). The docking results showed that the RNA_(50 nt)_ had three binding sites for VEGF. The strongest binding site was site 1, which included R23, P28, N100, P53, T77, E93, Q89, and H86 (**Figure** **6**A and Figure S7, Supporting Information). Based on the docking data, strong electrostatic interactions occurred between the positively charged amino acids and negatively charged phosphate groups of the RNA backbone. In contrast, RNA_(15 nt)_–VEGF had only one binding site, and its binding mode was similar to that of site 3 in RNA_(50 nt)_–VEGF (Figure 6B; Figure S8, Supporting Information). Taken together, RNA_(50 nt)_ demonstrated a stronger affinity for VEGF than RNA_(15 nt)_ (Table S2, Supporting Information).

An inhibition test was performed to confirm whether binding between the two molecules was electrostatic. RNase and a polycationic macromolecule (polyethyleneimine, PEI) were used to interfere with the binding between RNA and VEGF. Significantly fewer fluorescent aggregates were identified after treatment with RNase (*p* < 0.001, Figure S9, Supporting Information) or PEI (*p* < 0.001, Figure 6C–E).

Similar to VEGF, most neurovascular cytokines are polycationic (ExPASy ProtParam; https://web.expasy.org/protparam/).^[^ ^41^ ^]^ These included nerve growth factor (isoelectric point (pI) = 9.18), brain‐derived neurotrophic factor (pI = 8.91), fibroblast growth factor 23 (pI = 9.59), neurotrophin 3 (pI = 9.24), semaphorin 4d (pI = 8.04), netrin 1 (pI = 9.1), and slit guidance ligand 3 (pI = 7.95). The polycationic nature of these neurovascular cytokines suggests that they can also bind to RNA. Therefore, the online tool Predictor of Natural Disordered Regions (http://www.pondr.com/) was used to detect the intrinsic disorder propensities of these neurovascular factors, which represent their binding ability to RNA.^[^ ^42^ ^]^ The disorder scores of VEGF, fibroblast growth factor 23, and neurotrophin 3 were all higher than 0.5 (Figure 6F). Therefore, these three proteins have the potential to bind RNA.^[^ ^43^, ^44^ ^]^ These data suggest that exRNAs interact with other polycationic neurovascular cytokines in a manner analogous to that of VEGF.

### 2.5 Effect of RNA–VEGF Complexes on the Neurovascularization Effects of VEGF

**Figure 7:**
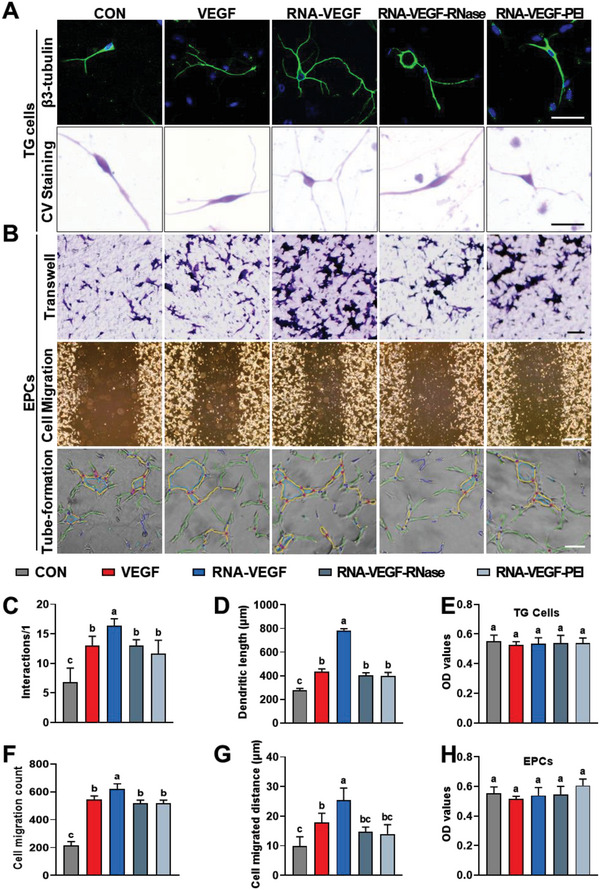
The exRNA‐VEGF complex stimulates angiogenesis and neurogenesis more than VEGF alone. A) Representative images of TG cells showing the length of their dendrites and their interactions after treatment for 24 h. B) Representative light microscopy images of EPCs showing cell migration and tube formation after treatment for 24 h. C,D) Quantification of dendritic length and interactions of TGs. E) CCK‐8 assay of the viability of TGs under the stimulation of different groups. F,G) Quantification of the numbers and distance of migrated EPCs. H) CCK‐8 assay of the viability of EPCs under the stimulation of different groups. Scale bars = 50 µm (A), 100 µm (top and bottom in B), and 500 µm (middle in B). Data are shown as the mean and standard deviation; *p* < 0.05 (*n* = 3). Different letters indicate statistically significant differences.

Different treatments were administered to primary TGs and EPCs to examine the effect of RNA–VEGF complexes on neurogenesis and angiogenesis. The morphology and axonal growth of TG cells were evaluated as indicators of neurogenesis, while EPC migration and tube formation were evaluated as indicators of angiogenesis (**Figure** **7**A,B). Compared to TG cells treated with VEGF only, cells treated with RNA–VEGF exhibited a longer dendritic length, more interactions, and a more elaborate morphology (*p* < 0.001). However, treatment with RNase or PEI significantly reduced the promoting effects of the RNA–VEGF complexes to the level observed in the VEGF‐only group (*p* < 0.001, Figure 7A,C,D). Treatment with RNase or PEI alone had no visually discernible effect on the morphology of TG cells compared to controls (*p* > 0.05, Figure S10, Supporting Information).

Similar to the effects of RNA–VEGF on neurogenesis, RNA‐VEGF complexes promote EPC migration and tube formation. The use of RNase or PEI significantly decreased the promoting effects of the RNA–VEGF complexes on the levels in the VEGF‐only group (*p* < 0.001, Figure 7B,F,G). Another form of RNA, 5s rRNA, was also found to bind to VEGF and promote neurogenesis, EPC migration, and tube formation (*p* < 0.05; Figure S11, Supporting Information). The cell proliferation assay indicated that the proliferation rates of TG (*p* > 0.05, Figure 7E) and EPCs (*p* > 0.05, Figure 7H) did not change significantly in any of the groups. These results indicate that RNA‐VEGF complexes amplify the neurovascularization effects of VEGF.

**Figure 8:**
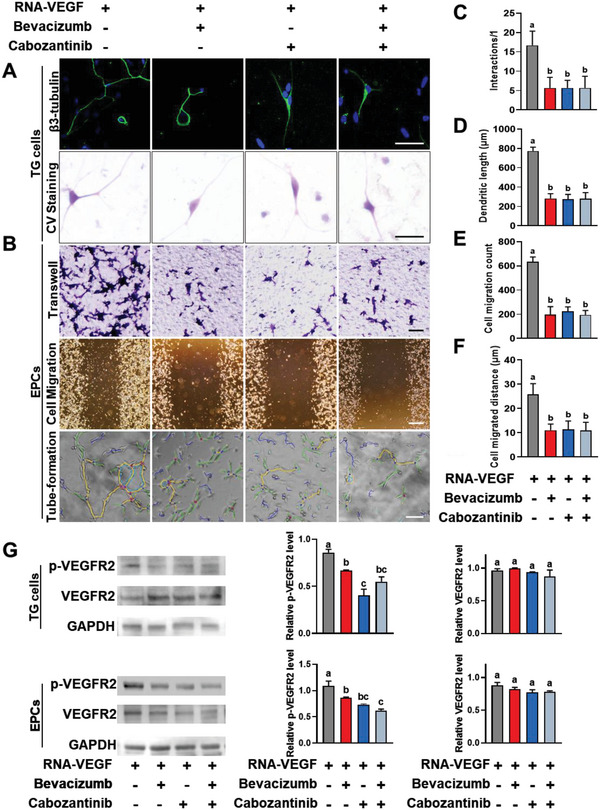
The exRNA‐VEGF complex promotes angiogenesis and neurogenesis through action on VEGFR2. A) Representative microscopy images of TG cells showing the dendritic length and interactions after 24 h treatment. B) Representative light microscopy images of EPCs showing migrated cells, cell migration, and tube formation after 24 h treatment. C,D) Quantification of dendritic length and interactions of TGs. E,F) Quantification of the numbers and distance of migrated EPCs. G) Western blot results showing expression of VEGFR2 and p‐VEGFR2 of TG cells and EPCs after treatment for 24 h. Scale bars = 50 µm (A), 100 µm (top and bottom in B), and 500 µm (middle in B). Data are shown as the means and standard deviations; *p* < 0.05 (*n* = 3). Different letters indicate statistically significant differences.

Vascular endothelial growth factor (VEGF) has three specific receptor tyrosine kinases; VEGFR1, VEGFR2, and VEGFR3. After receipt of VEGF signals, VEGFR2 molecules are modified to generate downstream signals for cell survival, migration, and proliferation.^[^ ^45^ ^]^ Unlike VEGFR2, the pro‐angiogenic effects of VEGFR1 and VEGFR3 are minimal. In the present study, to test whether RNA‐VEGF complexes promote neurovascularization via VEGFR2, VEGF antibodies (bevacizumab) or a VEGFR2 inhibitor (cabozantinib) were added to RNA‐VEGF complex‐treated EPCs and TG primary cells. The results showed that the pro‐neurovascular function of the RNA‐VEGF complex was significantly inhibited by both VEGF antibodies and the VEGFR2 inhibitor (*p* < 0.001, **Figure** **8**A–F).

**Figure 9:**
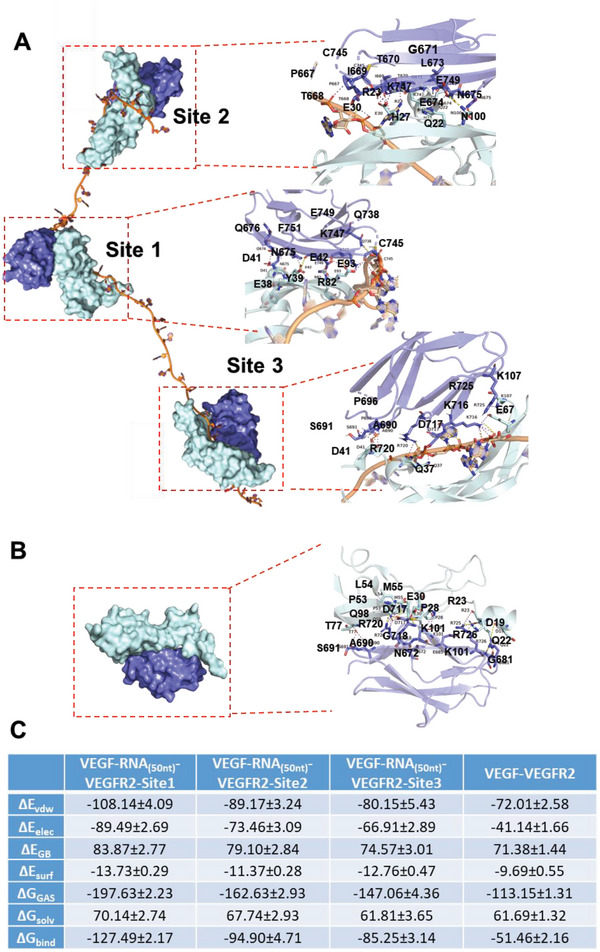
RNA_(50 nt)_ promotes binding between VEGF and the VEGFR2 extracellular segment. A) Binding pattern between RNA_(50 nt)_‐VEGF and VEGFR2 extracellular segment. B) Binding pattern between VEGF and VEGFR2 extracellular segment. The VEGFR2 extracellular segment is displayed as a blue ribbon, VEGF is displayed as a cyan ribbon, and RNA is displayed as an orange ribbon. C) Calculation of binding free energy between VEGFR2 extracellular segment and RNA‐VEGF complex/VEGF (kcal mol^−1^).

The binding mode between the RNA_(50 nt)_–VEGF complex and the VEGFR2 extracellular domain was further investigated by western blotting (Figure 8G) and molecular docking (**Figure** **9**). Consistent with the aforementioned results, site 1 had the strongest binding free energy for VEGFR2, followed by sites 2 and 3. All three sites of RNA_(50 nt)_‐VEGF firmly bound to VEGFR2 (Figure 9A–C; Figures S12 and S13, Supporting Information). These results indicated that binding with RNA enhanced the interaction between VEGF and VEGFR2. Analysis of the binding free energy showed that the major molecular interactions were electrostatic and van der Waals forces (Figure 9C). These results indicate that RNA promotes stronger binding between VEGF and VEGFR2. Furthermore, increased VEGFR2 phosphorylation was detected after stimulation with the RNA‐VEGF complex. This increase was disrupted by VEGF antibodies and VEGFR2 inhibitors (*p* < 0.001; Figure 8G). Further studies are required to explore the downstream signaling of enhanced VEGFR2 activation by RNA–VEGF complexes.

### 2.6 Effects of RNase and PEI on Neurovascularization, and Osteochondral Pathology

**Figure 10:**
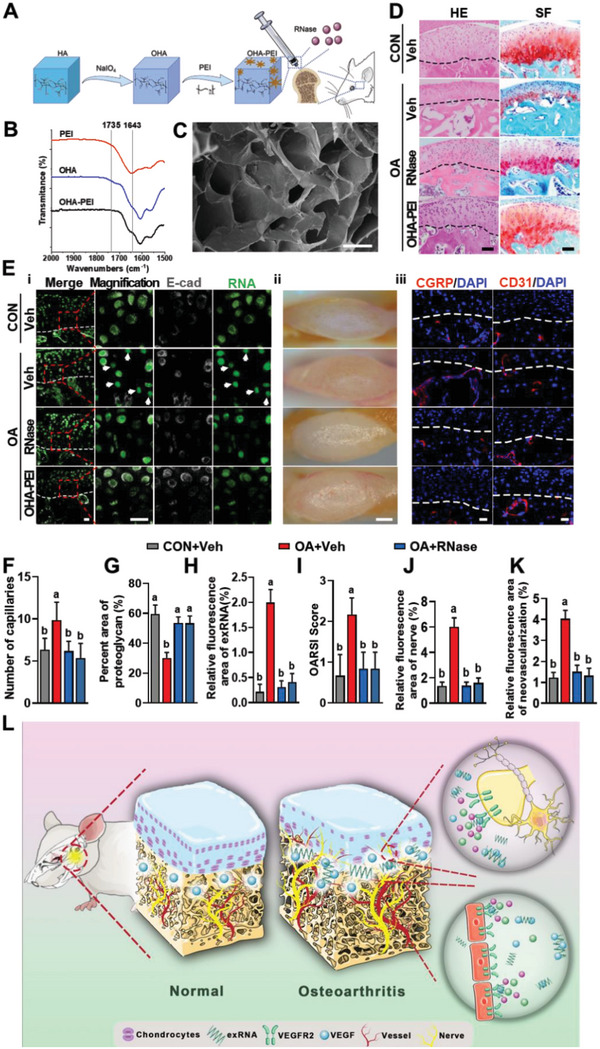
exRNA scavengers alleviate OA in murine TMJ. A) Schematic of the UAC mouse model and related interventions. B) Infrared spectra of OHA, PEI, and OHA‐PEI. C) SEM images of the surface of OHA‐PEI. D) Representative images of H&E and SF stainings of the condyles of the control (CON) and experimental (OA) groups at 3 weeks. E) Representative CLSM images of exRNA (i), macrophotographs (ii), nerves (CGRP, red), and vessels (CD31, red) (iii) after treatment. F–K) Statistical analysis of (D) and (E). L) Schematic summarizing the findings. During the progression of OA, exRNAs accumulated in the osteochondral junction. The exRNAs recruited positively‐charged neurovascular cytokines such as VEGF, and amplified the effect of VEGF on neurogenesis and angiogenesis. Veh referred to vehicle (i.e., saline). Scale bars = 70 µm (D), 1 mm (E, i), 20 µm (E, ii), 20 µm (E, iii). Data are shown as the mean and standard deviation; *p* < 0.05 (*n* = 3). Different letters indicated statistically significant differences.

RNase has been locally used to treat diseases such as cancer and inflammation.^[^ ^46^, ^47^ ^]^ To confirm the function of exRNA in vivo, RNase, PEI‐OHA hydrogel (details in the Experimental Section), and an equivalent volume of saline was injected intra‐articularly once per week into mice during the experimental period (**Figure** **10**A). The composition and surface topography of the PEI‐OHA hydrogels were analyzed using Fourier Transform Infrared Spectroscopy (Figure 10B) and scanning electron microscopy (Figure 10C). Treatment with RNase and PEI‐OHA significantly alleviated osteochondral pathologies (subchondral bone capillaries, proteoglycan content, and OARSI scores), abrogated excessive neurovascularization in osteoarthritic joints (*p* < 0.05, Figure 10D–K), and decreased the amount of exRNA (*p* < 0.001, Figure 10E,H). In contrast, clearance of exDNA by DNase did not reverse the osteochondral pathology or excessive neurovascularization (*p* > 0.05, Figure S14, Supporting Information). Taken together, these results indicate that exRNAs promote neurovascularization in the osteochondral junction by binding to VEGF. The clearance of exRNAs effectively inhibits neurovascularization and alleviates the progression of OA.

exRNAs are released into the extracellular matrix (where they are rapidly degraded) by living cells upon damage, or by cells undergoing apoptosis^[^ ^48^ ^]^ or autophagy.^[^ ^21^ ^]^ However, the degradation resistance of exRNAs increases in the presence of adequate RNA‐protein interactions or the formation of RNA–protein complexes. Owing to their increased resistance to degradation, the lingering time of exRNAs within the extracellular matrix can be prolonged.^[^ ^38^ ^]^ In the present study, exRNAs were derived from osteoarthritic chondrocytes or bone cells at the osteochondral junction. These cells undergo cell death and secrete exRNAs during OA progression.^[^ ^21^, ^48^, ^49^ ^]^ The released exRNAs interact with VEGF at the osteochondral junction. This protects the exRNAs from rapid degradation. In addition, the RNase content was reduced, and the RNase activity was decreased in osteoarthritic condyles, thereby enabling more exRNAs to survive in the extracellular matrix and promote neurovascularization. Although exDNA molecules were also present in the extracellular matrix,^[^ ^50^ ^]^ clearance of exDNA did not reverse osteochondral pathology or excessive neurovascularization. These results indicated that exRNA, but not exDNA, promotes neurovascularization and induces osteoarthritic changes.

These results may help to promote possible therapeutic approaches. We suggest that it may be possible to prevent excessive neurovascularization that exacerbates OA by blocking exRNAs in situ. Positively charged cationic materials have previously been used as exRNA scavengers for the treatment of inflammatory bowel disease,^[^ ^51^ ^]^ periodontitis,^[^ ^52^ ^]^ sepsis,^[^ ^53^ ^]^ and psoriasis.^[^ ^54^ ^]^ Such cationic materials could be employed to enhance VEGF function and treat diseases associated with abnormal neurovascularization. From a tissue‐engineering perspective, precise delivery of exRNA to lesions can stimulate localized VEGF function and neurovascularization. Developing cationic nanoparticles or hydrogels to achieve the controlled release of exRNA could promote soft and hard tissue repair by enhancing VEGF function and neurovascularization in the defect areas.

In summary, in the present study, we found that exRNAs have the ability to recruit polycationic neurovascular factors, such as VEGF. Recruitment of VEGF amplified its effect on abnormal neurogenesis and angiogenesis in the osteoarthritic condylar joint (Figure 10L). However, whether this mechanism is universal to other neurovascular factors requires further investigation. This study provides new insights into the mechanisms underlying neurovascularization, and further promotes the clearance of exRNAs via RNase or cationic nanoparticles as a prototype for the design of clinical strategies for treating neurovascularization‐related diseases.

## 3 Experimental Section

### Murine OA Model and In Vivo Injection

Twenty‐four female C57BL/6J mice (8 weeks old, 17–19 g) were provided by the Laboratory Animal Center of the Air Force Medical University (AFMU; Xi'an, Shaanxi Province, China). Only female mice were used in this study due to their higher morbidity rate. All experimental procedures, including animal experiments, were approved by the Institutional Ethics Committee of AFMU (No.2021‐001), in accordance with the “Animal Research: Reporting of In Vivo Experiments” guidelines for preclinical animal studies.

Mice were randomly divided into two groups: the unilateral anterior crossbite group (OA) and a sham‐operated control group (CON). Mice in the OA group were anesthetized with 1% intraperitoneal sodium pentobarbital. A unilateral anterior crossbite procedure was applied to the dentition of each mouse.^[^ ^21^, ^22^, ^23^, ^24^ ^]^ Briefly, pinheads (Shinva Ande, Shandong, China) were used to form 1.5 mm‐ and 5 mm‐long metal tubes. The shorter incisors were adhered to the left maxillary incisor with zinc phosphate cement. The longer tooth was bent at an angle of 135 ° at the end of the tube and was adhered to the left mandibular incisor. The mice in the CON group were subjected to the same procedure, but without metal tube fixation.

For TMJ injection, each mouse was lightly anesthetized using a mixture of 2% isoflurane and oxygen. Mice were then deeply anesthetized with pentobarbital and placed on one side. A needle with a custom‐designed Hamilton syringe was used to puncture the zygomatic arch between the corners of the eye and ear. This enabled the needle to slide along the bony wall and reach the TMJ. RNase (10 ng µL^−1^, dissolved in 50 µL saline, Thermo Fisher Scientific, Waltham, MA), PEI‐OHA hydrogel (50 µL), and saline (50 µL) was injected locally into the TMJ region weekly for three consecutive weeks. The first injection was administered immediately after the placement of the unilateral anterior crossbite appliance.

Injection procedures for Trigeminal ganglion inoculation and anterograde tracing were performed.^[^ ^55^ ^]^ A recombinant self‐fluorescent adeno‐associated virus, rAAV‐CAG‐mCherry‐WPRE‐hGH polyA (BrainVTA, Wuhan, China; 2×10^12^ viral genomes/mL), was used for inoculation. Each mouse to be inoculated was lightly anesthetized under 2% isoflurane and oxygen and then deeply anesthetized with pentobarbital. A midline skin incision (2 cm long) was made from the head to the neck to expose the cranium. A craniotomy (1 mm in diameter) was performed over the bregma region using a dental drill. A glass micropipette (tip diameter 40–60 µm) connected to a microsyringe (1 µL, Hamilton, NV, USA) was inserted into the target site for delivery of adeno‐associated virus (100 nL). After the inoculation, the wound was sutured. After 1 week, the mice were randomly divided into the osteoarthritic group and CON groups.

All mice were euthanized by pentobarbital overdose 3 weeks after insertion of the unilateral crossbite appliance. Some of condyles were harvested and fixed with 4% paraformaldehyde at 4 °C for 24 h. The fixed condyles were decalcified with 4% ethylenediamine tetraacetic acid solution for 4 weeks at room temperature, dehydrated in 30% sucrose for 3 days, embedded in optimal cutting temperature compound (Leica, Wetzlar, Germany), and stored at −80 °C. Central sagittal sections were prepared using a cryostat‐supported microtome (CM1950; Leica, Weitzlar, 692 Germany). Some of the condyles were fixed in 2.5% glutaraldehyde at 4 °C for scanning electron microscopy (SEM), and transmission electron microscopy (TEM), while other were immediately used for morphological observation using a stereoscopic microscope. The rest of the condyles were preserved at −80 °C for qRT‐PCR and exRNA isolation.

### Histochemical and Immunofluorescence Staining

For histological staining, central sagittal sections of each condyle were stained with hematoxylin and eosin (HE), safranin O/fast green, or glycine silver. Stained sections (*n* = 3) were examined under a light microscope (Leica Microsystems, Wetzlar, Germany) and analyzed using ImageJ software (National Institutes of Health, Bethesda, MD, USA).

For immunofluorescence staining, the central sagittal sections of each condyle were treated with 1% Triton X‐100 (MilliporeSigma, Burlington, MA, USA), blocked with 10% goat serum (Beyotime, China), and incubated with primary antibodies at 4 °C overnight. The primary antibodies used were EMCN (1:300, sc‐65495, Santa Cruz Biotechnology, Dallas, TX, USA), CD31 (1:300, sc‐376764, Santa Cruz Biotechnology), PGP 9.5 (1:300, ab8189, Abcam, Cambridge, United Kingdom), CGRP (1:400, 14959, Cell Signaling Technology, Danvers, MA, USA), *α*‐tubulin (1:300, 2144, Cell Signaling Technology), E‐cadherin (1:300, ab231303, Abcam), and VEGF (1:300, sc‐7269, Santa Cruz Biotechnology). Sections were washed with phosphate‐buffered saline (PBS), and incubated with a secondary antibody for 1 h in the dark. The corresponding secondary antibodies (1:400; ab150117, ab150080, ab150116, and ab150083; Abcam) were used. The sections were then mounted with Prolong Diamond Antifade Mountant containing 4′,6‐diamidino‐2‐phenylindole (DAPI) (S2110, Solarbio, China), and observed using confocal laser scanning microscopy (CLSM; Nikon A1R, Nikon Corporation, Minato‐ku, Tokyo, Japan). Three randomly selected nonoverlapping viewing fields were obtained for each section. The relative fluorescence area was analyzed semi‐quantitatively using ImageJ software. exRNAs were defined as any flocculently arranged RNAs distant from E‐cadherin, *α*‐tubulin, and DAPI.^[^ ^18^ ^]^ The steps for exRNA quantification are summarized in Figure S15 (Supporting Information).^[^ ^56^ ^]^

### SEM and TEM

Condyles fixed in 2.5% glutaraldehyde at 4 °C were used for Scanning electron microscopy (SEM; JSM‐6701F, JEOL, Tokyo, Japan) and transmission electron microscopy (TEM; H‐600, Hitachi, Tokyo, Japan) to examine the ultrastructural changes at the osteochondral junction. Tissue processing was performed according to the standard procedures.^[^ ^21^ ^]^

### Quantitative Real‐Time Polymerase Chain Reaction (qRT‐PCR)

qRT‐PCR was performed as previously described.^[^ ^21^ ^]^ The expression levels of platelet‐derived growth factor subunit B (*Pdgfb*), nerve growth factor (*Ngf*), vascular endothelial growth factor A (*Vegf*), hypoxia‐inducible factor 1 subunit alpha (*Hif1α*), hypoxia‐inducible factor 2 subunit alpha (*Hif2α*), netrin 1 (*Ntn1*), netrin 3 (*Ntn3*), netrin 4 (*Ntn4*), slit guidance ligand 1 (*Slit1*), slit guidance ligand 2 (*Slit2*), and slit guidance ligand 3 (*Slit3*) were examined. Glyceraldehyde‐3‐phosphate dehydrogenase (*Gapdh*) was used as a housekeeping gene. The primer sequences are listed in Table S3 (Supporting Information). Statistical analysis was performed in GraphPad Prism 8.0 using the 2^–∆∆Ct^ method. Student's *t*‐test was used for statistical comparisons. Statistical significance was preset *α* = 0.05 (*n* = 3).

### Quantitative Analysis of RNase Concentration and Activity

Condyles isolated from the CON and OA groups were frozen, crushed in liquid nitrogen, and soaked in RIPA Lysis and Extraction Buffer (50 µL, Thermo Fisher Scientific) for 15 min on ice. After centrifugation (12 000 rpm, 15 min, 4 °C), the supernatant was used to quantify RNase concentration and activity using a sandwich ELISA assay (Cloud Clone, Wuhan, China), and the Ambion RNaseAlert QC system (*n* = 3).

### ExRNA Isolation and Characterization

Condyles isolated from the control and experimental groups were frozen and shattered in liquid nitrogen, soaked in diethylpryrocarbonate‐treated water (50 µL), and placed in a shaker (50–80 rpm) for 1 week at 4 °C. exRNA was isolated and concentrated using MagMAX mirVana Total RNA Isolation Kit (ThermoFisher Scientific).^[^ ^57^ ^]^ The exRNA extracted from one condyle was collected in elution buffer (10 µL), and quantified using NanoDrop 2000 ultraviolet spectroscopy (ThermoFisher Scientific).

### Inductively Coupled Plasma–Mass Spectrometry

The exRNA solutions and their vehicle (Elution Buffer in MagMAX mirVana Total RNA Isolation Kit) were digested in nitric acid (10 mL) and acid mixture (10 mL, hydrofluoric acid: perchloric acid = 10:1) at 230 °C for 4 h. After drying, aqua regia (10 mL) and distilled water (15 mL) were added and the solution was diluted. Chemical analysis of the exRNA solutions was performed using inductively coupled plasma‐mass spectrometry (Aglient 7800) and analyzed using Graphpad Prism 8.0 (*n* = 3).

### Microarray Statistics

The gene expression profile of GSE45793 was obtained from the NCBI for Biotechnology Information Gene Expression Omnibus (http://www.ncbi.nlm.nih.gov/geo/). This profiling was previously performed using a GPL4134 Agilent‐014868 Whole Mouse Genome Microarray 4 × 44 K G4122F. GSM1120025‐GSM1120028 was used for the OA group. GSM1120044‐GSM1120047 were used as the control groups. Differentially expressed genes between the two groups were identified using the GEO2R online analysis tools. Neurovascular cytokines were selected and their fold changes are shown in Table S1 (Supporting Information).

### Binding of RNA to VEGF In Vivo

Osteoarthritic condyles were frozen and crushed in liquid nitrogen, soaked in RIPA Lysis (20 µL, Thermo Fisher Scientific) for 15 min on ice. After centrifugation (15 000 rpm, 15 min, 4 °C), the supernatant was incubated with biotin‐labeled VEGF antibody and streptavidin coupled magnetic beads (Dynabeads M‐280 Streptavidin, Invitrogen) overnight. After PBSP (0.02% Tween‐20 in PBS) washing, the magnetic beads were eluted with 10 µL 0.1 m Glycine (pH 2.0). Eluate containing the VEGF‐antibody affinity purified complex (named “VEGF solution” group), and the pure eluate (that is the 0.1 m Glycine solution, named “Veh” group), were tested for the VEGF protein by western blot, and for RNA by the NanoDrop test.

### Binding of RNA to VEGF In Vitro

Microtiter plate wells were coated with VEGF (50 µL, 150 µg mL^−1^, 0311199, Pepro Tech, Offenbach, Germany) in 100 mm sodium carbonate (pH 9.5) at 4 °C. The wells were washed and blocked with Tris‐buffered saline supplemented with 3% bovine serum albumin (MilliporeSigma). RNA_(15 nt)_ (50 µL, 150 µg mL^−1^, CY3‐AAAAAAAAAAAAAAA, Sangon, Shanghai, China), or RNA_(50 nt)_ (50 µL, 150 µg mL^−1^, CY3‐AAAAAAAAAAAAAAAAAAAAAAAAAAAAAAAAAAAAAAAAAAAAAAAAAAAA, Sangon), or synthetic rRNAs (prepared by transcription from synthetic DNA templates, NCBI Reference Sequence: XR_004940552.1), were incubated at 22 °C for 2 h. Bound RNA_(15 nt)_ and bound RNA_(50 nt)_ were detected using CLSM by measuring Cy3 fluorescence signals.^[^ ^58^ ^]^ For the inhibition experiment, RNA_(50 nt)_ was pre‐incubated with RNase (10 µL, 1 µg mL^−1^), or polyethyleneimine (10 µL, 1 µg mL^−1^), at room temperature prior to adding it to the protein‐coated wells. The results were analyzed using GraphPad Prism 8.0 (*n* = 3).

### In Vitro Binding Assay

Recombinant VEGF was labeled with Alexa Fluor 488 (Thermo Fisher Scientific). Alexa Fluor 488‐labeled VEGF (20 µL 150 µg mL^−1^) was mixed with RNA_(50 nt)_ (20 µL, Cy3‐labeled, 150 µg mL^−1^) in 96‐well plates coated with bovine serum albumin. After incubation, images were captured at the indicated times using CLSM to detect Alexa Fluor 488 and Cy3 fluorescence.^[^ ^59^ ^]^ Results were analyzed using GraphPad Prism 8.0 (*n* = 3).

### Size Distribution and Zeta Potential Measurement

The size distribution and zeta potential were measured using a Litesizer 500 particle analyzer (Anton Paar, Graz, Austria). Three entities were examined: VEGF (150 µg mL^−1^), RNA_(50 nt)_ (150 µg mL^−1^), and VEGF ‐ RNA_(50 nt)_ (150 µg mL^−1^). The results were analyzed using GraphPad Prism 8.0 (*n* = 3).

### RNA‐VEGF Degradation Assay

Agarose nucleic acid electrophoresis on 2% agarose gels containing TBE buffer was applied to detect the degradation of RNA or RNA‐VEGF complexes. Synthetic RNA (500 ng µL^−1^) and VEGF (500 ng mL^−1^) were incubated for 2 h at room temperature. RNase (1 g mL^−1^) was further added and incubated for 1 h at 37 °C. The electrophoresis bands and their positions were observed using an Azure 600 (Azure Biosystems, CN) (*n* = 3).

### Endothelial Progenitor Cells and Primary Culture of Trigeminal Ganglion Neurons

For culture of endothelial progenitor cells (EPCs; Jennio Biotech, Guangzhou, Guangdong, China), the control medium consisted of Dulbecco's modified Eagle's medium (Hyclone, Logan, UT, USA), 10% fetal bovine serum (Gibco, Thermo Fischer Scientific), and 1% penicillin/streptomycin (1:100, MilliporeSigma). Culture was incubated in 5% CO_2_ at 37 °C. The medium was changed every other day. The EPCs were divided into different groups: no treatment, VEGF (2.5 ng mL^−1^), RNA (2.5 ng mL^−1^), RNA–VEGF complex (2.5 ng mL^−1^), RNase (1 µg mL^−1^), PEI (1 µg mL^−1^), RNA–VEGF (2.5 ng mL^−1^)‐RNase (1 µg mL^−1^), RNA‐VEGF (2.5 ng mL^−1^)‐PEI (1 µg mL^−1^), RNA‐VEGF (2.5 ng mL^−1^)‐bevacizumab (250 ng mL^−1^), RNA‐VEGF (2.5 ng mL^−1^)‐cabozantinib (1 µm), RNA‐VEGF (2.5 ng mL^−1^)‐bevacizumab (250 ng mL^−1^)‐cabozantinib (1 µm). The RNA used was RNA_(50 nt)_.

EPCs were subjected to a cell scratch test. For this test, cells were incubated with fetal bovine serum (free culture medium only) or different conditioned media for 24 h. The transwell cell migration assay was performed according to a previously reported protocol.^[^ ^60^ ^]^ The bottom surfaces of the transwells were stained with crystal violet for 24 h to observe cell migration. Tube formation assays were performed according to manufacturer's instructions (Corning).^[^ ^61^ ^]^ Three randomly selected fields from all groups were captured after 24 h using an inverted microscope (Leica, Mannheim, Germany). Data were analyzed using the ImageJ software (GraphPad Prism 8.0; *n* = 3).

For primary culture of trigeminal ganglion neurons, neonatal Sprague‐Dawley rats (1–5 days old) were decapitated, and their trigeminal ganglia were aseptically dissected. The isolated neurons were digested with 0.1% collagenase and 0.25% trypsin (ThermoFisher Scientific). After centrifugation (1000 rpm, 3 min), the pellet was resuspended and plated at a density of approximately 1.0 × 10^3^ cells on poly‐d‐lysine hydrobromide (50 ng mL^−1^, Thermo Fisher Scientific)‐coated cell culture plates. Neurobasal media (Invitrogen), containing 0.5 mm l‐glutamine, penicillin/streptomycin (1:100, MilliporeSigma), and 2% B‐27 (Invitrogen), was used as the culture medium. Culture was incubated in 5% CO_2_ at 37 °C. The medium was changed every other day. Four hours after culture, the TG neurons were divided into the 11 groups mentioned above for EPCs The RNA used and RNA_(50 nt)_. Crystal violet and immunofluorescence staining of *β*3‐tubulin (1:300, ab18207, Abcam) were used to evaluate the axonal morphology of TG neurons (*n* = 3).

The cell viability of EPCs and TGs was estimated using the CCK‐8 assay kit (*n* = 3; Dojindo Molecular Technologies, Rockville, MD, USA), and a cell medium without cells was used as a negative control.

### Molecular Docking

X‐ray crystal structure files of VEGF and VEGFR2 were obtained from the PDB Data Bank (PDB ID:1VPF and 3KVQ, respectively). The structures of RNA_(15 nt)_ and RNA_(50 nt)_ were prepared using the RNAfold online server, RNA Composer, and AmbrTools, based on their sequences. Prior to molecular docking, all water molecules were removed and nonpolar hydrogen atoms were added using UCSF Chimera. The AutoDock 4.2 software ( Scripps Research Institute, La Jolla, CA, USA) was used for the docking analysis. The docking volume was preset as 60 Å × 60 Å × 60 Å. The positions and orientations of the ligands were determined by genetic algorithms. In the present study, “molecular mechanics with generalized born and surface area solution” (MM/GBSA)^[^ ^62^ ^]^ was used to calculate the binding free energy of nucleic acid molecules and the Gibbs binding energy (ΔG_bind_) of the ligands. The MMPBSA.py program^[^ ^63^ ^]^ integrated with AmberTools was used to calculate the binding energy using the following formula:

(1) $${\Delta G}_{\mathrm{bind}}=\Delta H-T\Delta S\approx {\Delta G}_{\mathrm{solv}}+{\Delta G}_{\mathrm{GAS}}-T\Delta S$$

(2) $${\Delta G}_{\mathrm{GAS}}=\Delta E_{\mathrm{int}}+\Delta E_{\mathrm{vdw}}+\Delta E_{\mathrm{elec}}$$

(3) $${\Delta G}_{\mathrm{solv}}=\Delta E_{\mathrm{surf}}+\Delta E_{\mathrm{GB}}$$

where ∆*G*_GAS_ represents the difference in kinetic energy in the vacuum before and after the binding of the receptor and ligand. This entity is subdivided into ∆*E*_int_, Δ*E*_vdw_, and Δ*E*_elec_, where ∆*E*_int_ represented the change in bond energy, bond angle, and dihedral angle; Δ*E*_vdw_ represented the change in van der Waals energy before and after binding; Δ*E*_elec_ represented the change in electrostatic action. Δ*G*_solv_ is the solvent effect term, which is divided into the polar term *E*_GB_ and the nonpolar term *E*_surf_. Calculation of ∆*E*_GB_ was performed using the APBS program. *E*_surf_ was calculated from the accessible surface area of the solution.

### Prediction of Isoelectric Point and Intrinsically Disordered Regions of a Protein

The pI of a protein is the pH at which the net protein charge is zero. A pI value higher than 7 indicates an alkaline protein, whereas a pI value lower than 7 indicates an acidic protein. Intrinsically disordered regions (IDRs) are polypeptide segments that do not contain enough hydrophobic amino acids to facilitate co‐operative folding. These segments were more likely to contain polar or charged amino acids. The ExPASy ProtParam online tool (https://web.expasy.org/protparam/) and the natural disordered region predictor (PONDR, http://pondr.com/)^[^ ^43^ ^]^ were used to predict pI and IDRs, respectively. Protein sequences were obtained from the UniProt database (https://www.uniprot.org/). Predictions were made for VEGF, BSA, NGF, BDNF, FGF23, NTF3, SEMA4d, NTN1, and Slit3.

### Western Blot

Western blot was performed to determine the relative expression or phosphorylation of VEGFR2 in TG cells and EPCs after 24 h of treatment (*n* = 3). A Pierce BCA protein assay kit (Solarbio, Beijing, China) was used to determine total protein concentration. Protein samples were separated on a 10% SDS‐polyacrylamide gel and transferred onto a polyvinylidene difluoride membrane (EMD Millipore Corp., Billerica, MA, USA). The membranes were blocked with 5% BSA and incubated for 2 h. The primary antibodies used were VEGFR2 (1:2000; 26415‐1‐AP; Proteintech, Chicago, IL, USA), and p‐VEGFR2 (1:2000; AF3279; Affinity Biosciences, Cincinnati, OH, USA). Signals were detected after incubation with horseradish peroxidase‐conjugated secondary antibody (Santa Cruz Biotechnology) and enhanced chemiluminescence detection (GeneTex, Irvine, CA, USA). The stained bands were scanned and quantified using a densitometer (Tanon, Shanghai, China) and the ImageJ software. The protein expression levels were normalized to those of GAPDH.

### Synthesis and Characterization of OHA‐PEI

1 g sodium hyaluronate (2.5 mmol) was dissolved in water (100 mL) at RT. A sodium periodate solution (0.982 mmol) was added dropwise to the dissolved hyaluronate, after which 500 µL ethylene glycol was added while stirring for 1 h. The reaction mixture (i.e., oxidized hyaluronic acid, OHA) was purified by dialysis, lyophilized, and stored at 4 °C. OHA was dissolved in distilled water, and the PEI solution was added. The final mass ratio was 1:1. The reaction mixture (i.e., OHA‐PEI) was lyophilized and stored at 4°C until use. The synthesis of OHA‐PEI was determined using attenuated total reflection‐Fourier transform infrared spectroscopy (ATR‐FTIR, Shimadzu 8400S, Shimadzu 605 Corp., Kyoto, Japan). Spectra were taken from 4000 to 400 cm^−1^, using 32 scans at a resolution of 4 cm^−1^. Spectral analysis was performed using IR solution software (Shimadzu). The surface topography of OHA‐PEI was examined by SEM.

### Statistical Analyses

Data obtained from each experiment were tested for normality and equal variance assumptions, using the Shapiro–Wilk and modified Levene tests respectively, prior to the use of parametric statistical methods. If either of these assumptions was violated, the corresponding datasets were nonlinearly transformed to satisfy them. All quantitative data were presented as mean ± standard deviation. Differences between two groups were tested using Student's *t*‐test. Differences between more than two groups were tested using one‐way analysis of variance (ANOVA and Tukey's multiple comparison tests. Pearson's correlation analysis was used to compare the correlations between the two groups. GraphPad Prism (version 8.0) was used for all the statistical analyses. For all tests, statistical significance was preset at *α* = 0.05.

## Conflict of Interest

The authors declare no conflict of interest.

## Supporting information

Supporting Information

Supplemental Table 1

## Data Availability

The data that support the findings of this study are available from the corresponding author upon reasonable request.
