# Supplementary material for: Effect of Extracellular Ribonucleic Acids on Neurovascularization in Osteoarthritis
Source: Adv Sci (Weinh). 2023 Jul 3;10(26):2301763. doi: 10.1002/advs.202301763 (PMC10502862; doi:10.1002/advs.202301763)
Supplement: Supplementary file 1 — Supporting Information [file ADVS-10-2301763-s001.pdf]

ADVANCED SCIENCE
Open Access

Supporting Information

for *Adv. Sci.*, DOI 10.1002/advs.202301763

Effect of Extracellular Ribonucleic Acids on Neurovascularization in Osteoarthritis

Wen-pin Qin, Qian-Qian Wan, Jian-Fei Yan, Xiao-Xiao Han, Wei-Cheng Lu, Zhang-Yu Ma, Tao Ye, Yu-Tao Li, Chang-Jun Li, Chen Wang, Franklin R. Tay, Li-Na Niu* and Kai Jiao**

Supporting Information

The effect of extracellular ribonucleic acids on neurovascularization in osteoarthritis

Wen-pin Qin, Qian-qian Wan, Jian-fei Yan, Xiao-xiao Han, Wei-cheng Lu, Zhang-yu Ma, Tao Ye, Yu-tao Li, Chang-jun Li, Chen Wang, Franklin R Tay, Li-na Niu*, Kai Jiao**

This SI file includes:

Figure S1. RNase level and activity are decreased in OA condyles than the controls.
Figure S2. Local RNA is adjacent to CGRP$^+$ cells.
Figure S3. Local RNA is adjacent to CD31$^+$ cells.
Figure S4. Local RNA is co-localized with VEGF.
Figure S5. The isolated solution contains VEGF.
Figure S6. RNA–VEGF complex prevents RNA from degradation.
Figure S7. Binding pattern between $RNA_{(50nt)}$ and VEGF.
Figure S8. Binding pattern between $RNA_{(15nt)}$ and VEGF.
Figure S9. RNase disturbs $RNA_{(50nt)}$-VEGF complex.
Figure S10. RNase or PEI along doesn't have inhibitory effect on the function of TG cells and EPCs.
Figure S11. rRNA have the similar effect on the function of VEGF.
Figure S12. Binding pattern between $RNA_{(50nt)}$-VEGF and VEGFR2 extracellular segment.
Figure S13. Binding pattern of VEGF and VEGFR2 extracellular segment.
Figure S14. DNase doesn't reverse the progression of OA.
Figure S15. The steps of exRNA quantification in Figure 2 sections.
Table S2. Calculation of binding free energy between VEGF protein and RNA (kcal/mol).
Table S3. Primer sequences used for qRT-PCR in the present study.

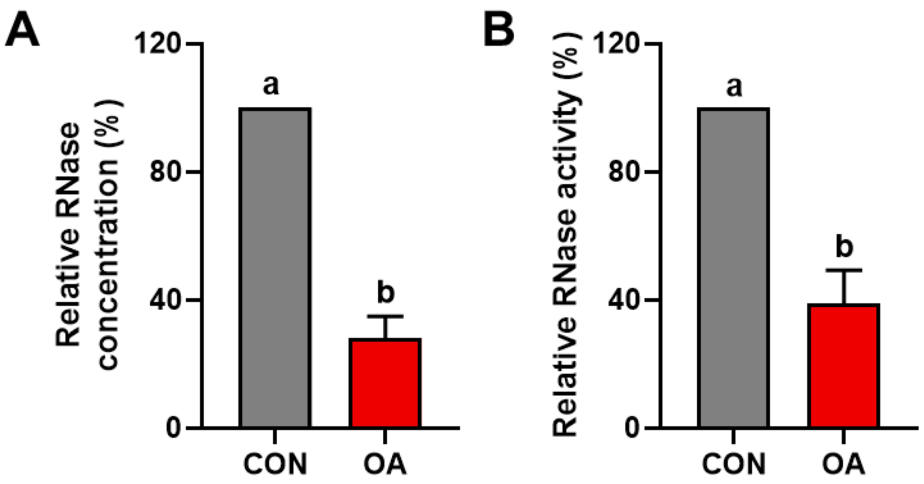

Figure S1. RNase level and activity are decreased in OA condyles than the controls. A The relative RNase concentration of OA condyles was decreased compared to CONs. **B** The relative RNase activity of OA condyles was decreased compared to CONs. Data are shown as the means and standard deviations; $p < 0.05$ ($n = 3$). Equivalent means have the same letter; different letters indicate statistically significant differences.

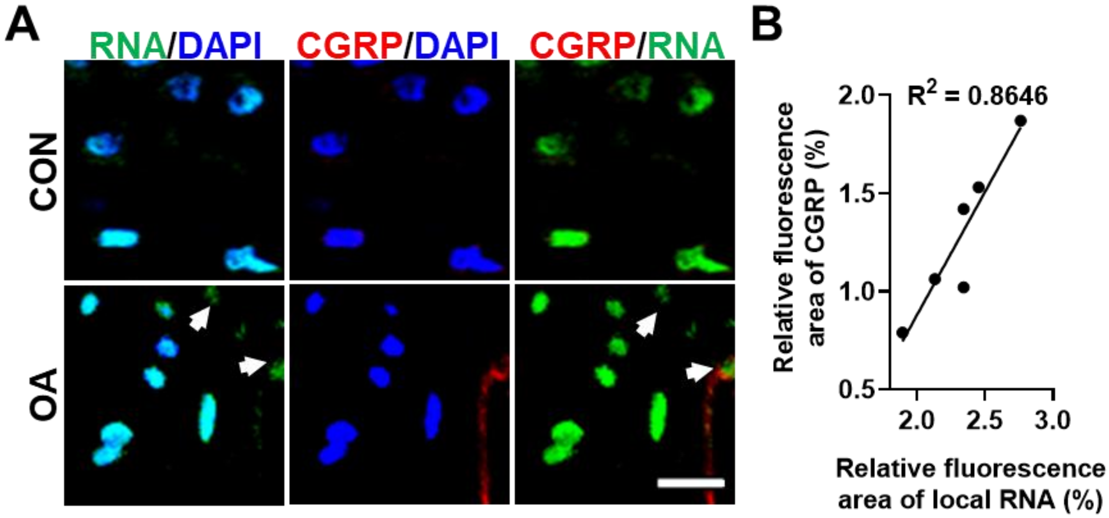

Figure S2. Local RNA is adjacent to CGRP+ cells. A CLSM images taken from osteochondral junction showing that local RNA is adjacent to nerves (CGRP, red). Arrows indicate local RNA and arrow heads represent the nerves. B Pearson correlation analysis of the relative fluorescence area of CGRP and local RNA (n = 6, R^2= 0.8646, p = 0.0072). Equivalent means have the same letter; different letters indicate statistically significant differences.

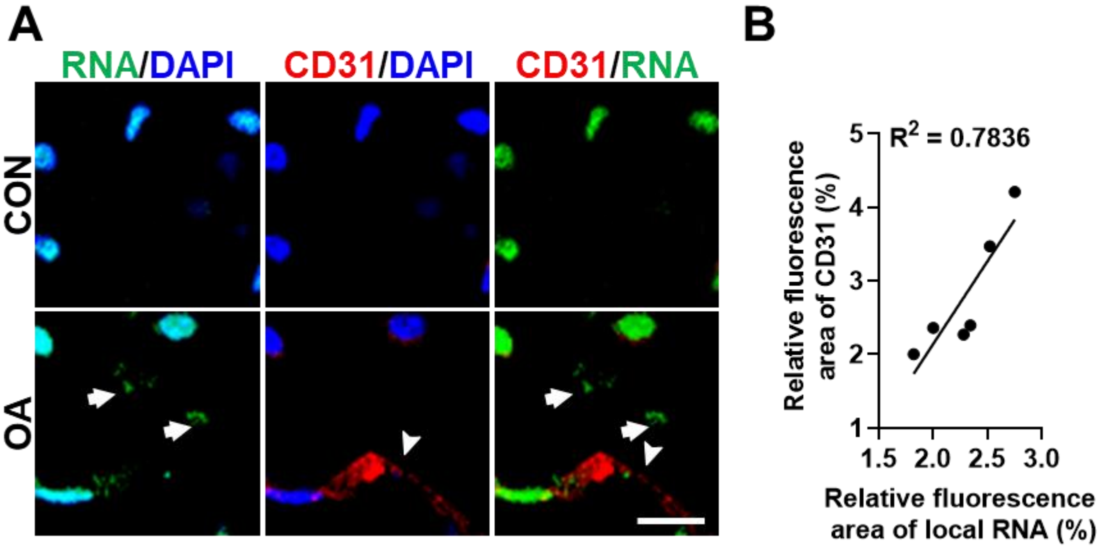

Figure S3. Local RNA is adjacent to CD31⁺ cells. A CLSM images taken from osteochondral junction showing that local RNA is adjacent to the newborn vessels (CD31, red). Arrows indicate local RNA and arrow heads represent newborn vessels. **B** Pearson correlation analysis of the relative fluorescence area of CD31 and local RNA (*n* = 6, R^2= 0.7836, *p* = 0.0190). Equivalent means have the same letter; different letters indicate statistically significant differences.

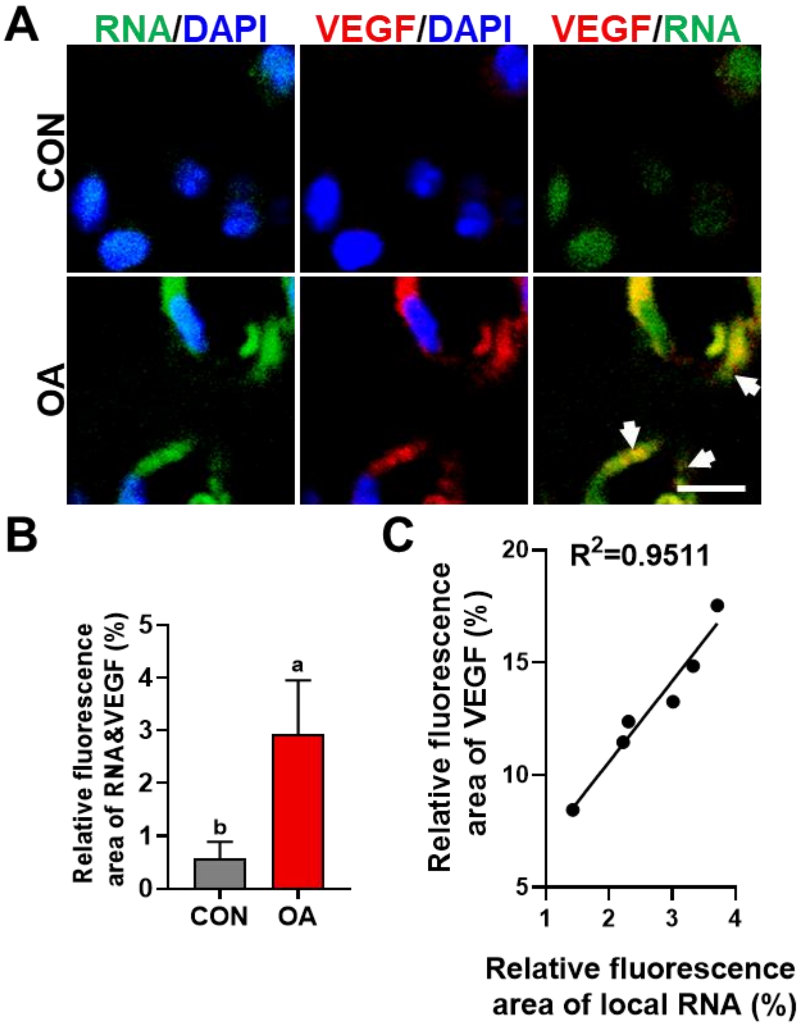

Figure S4. Local RNA is co-localized with VEGF. A CLSM images taken from osteochondral junction showing that local RNA is co-localized with VEGF (red). Arrows indicate co-localization of local RNA and VEGF. **B** The relative fluorescence area of the co-localization of local RNA and VEGF (**A**). **C** Pearson correlation analysis of the relative fluorescence area of CD31 and local RNA (n = 6, R^2= 0.7836, p = 0.0190). Data are shown as the means and standard deviations; $p < 0.05$ (n = 3). Equivalent means have the same letter; different letters indicate statistically significant differences.

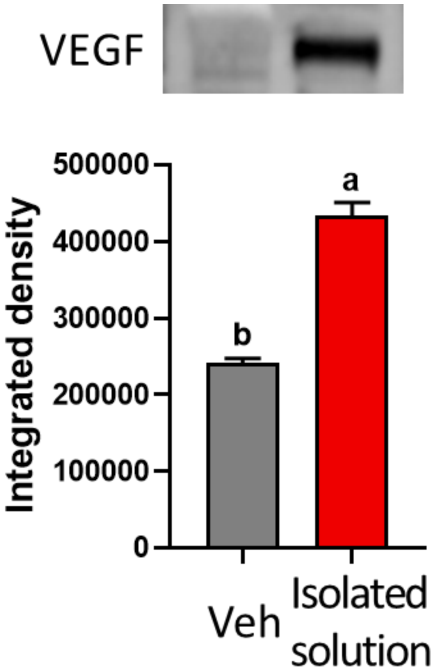

Figure S5. The isolated solution contains VEGF. Western Blot is used to detect the existence of VEGF in eluate containing the VEGF-antibody affinity purified complex. Veh refers to vehicle (i.e., the 0.1M glycine solution). Data are shown as the means and standard deviations; $p < 0.05$ ($n = 3$). Equivalent means have the same letter; different letters indicate statistically significant differences.

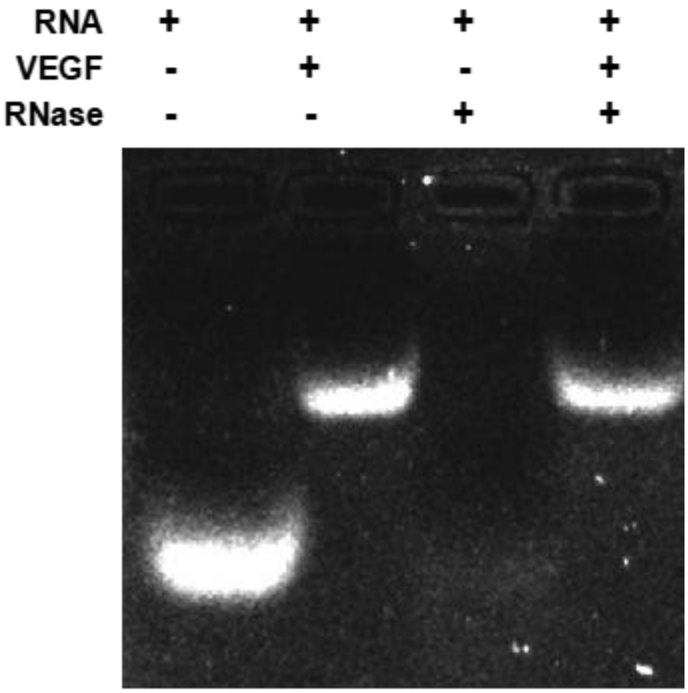

Figure S6. RNA–VEGF complex prevented RNA from degradation. Agarose gel electrophoresis depicts RNA is more stable after binding with VEGF.

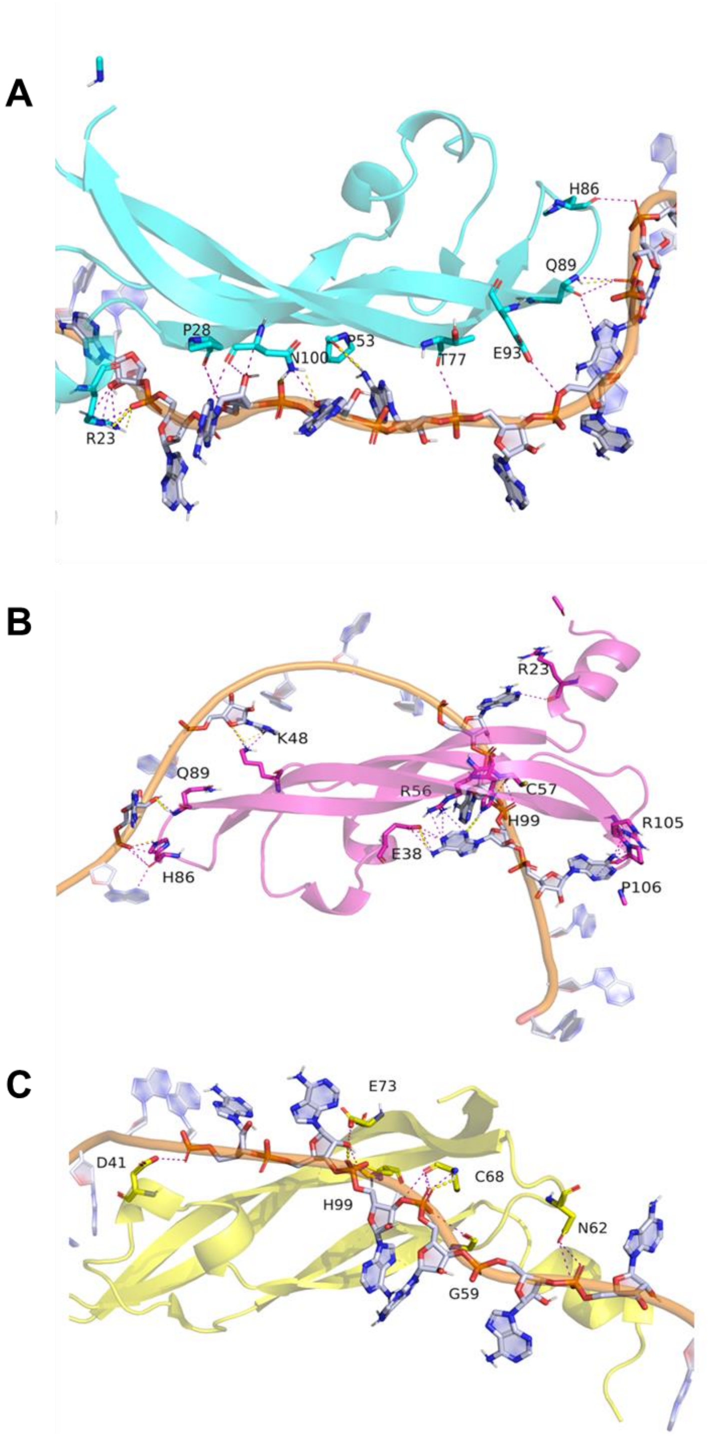

Figure S7. Binding pattern between RNA$_{(50nt)}$ and VEGF. A, B, and C represents Site 1, Site2, and Site 3 respectively. Hydrogen bonds are shown as yellow dotted lines, and van der Waals contacts within 4 Å are shown as magenta dotted lines. The O atom is red, the N atom is blue, the H atom is white, and the S atom is yellow. Protein uses ribbon display mode and RNA uses transparent surface display mode and stick display mode. PyMOL software is used to draw pictures and interact.

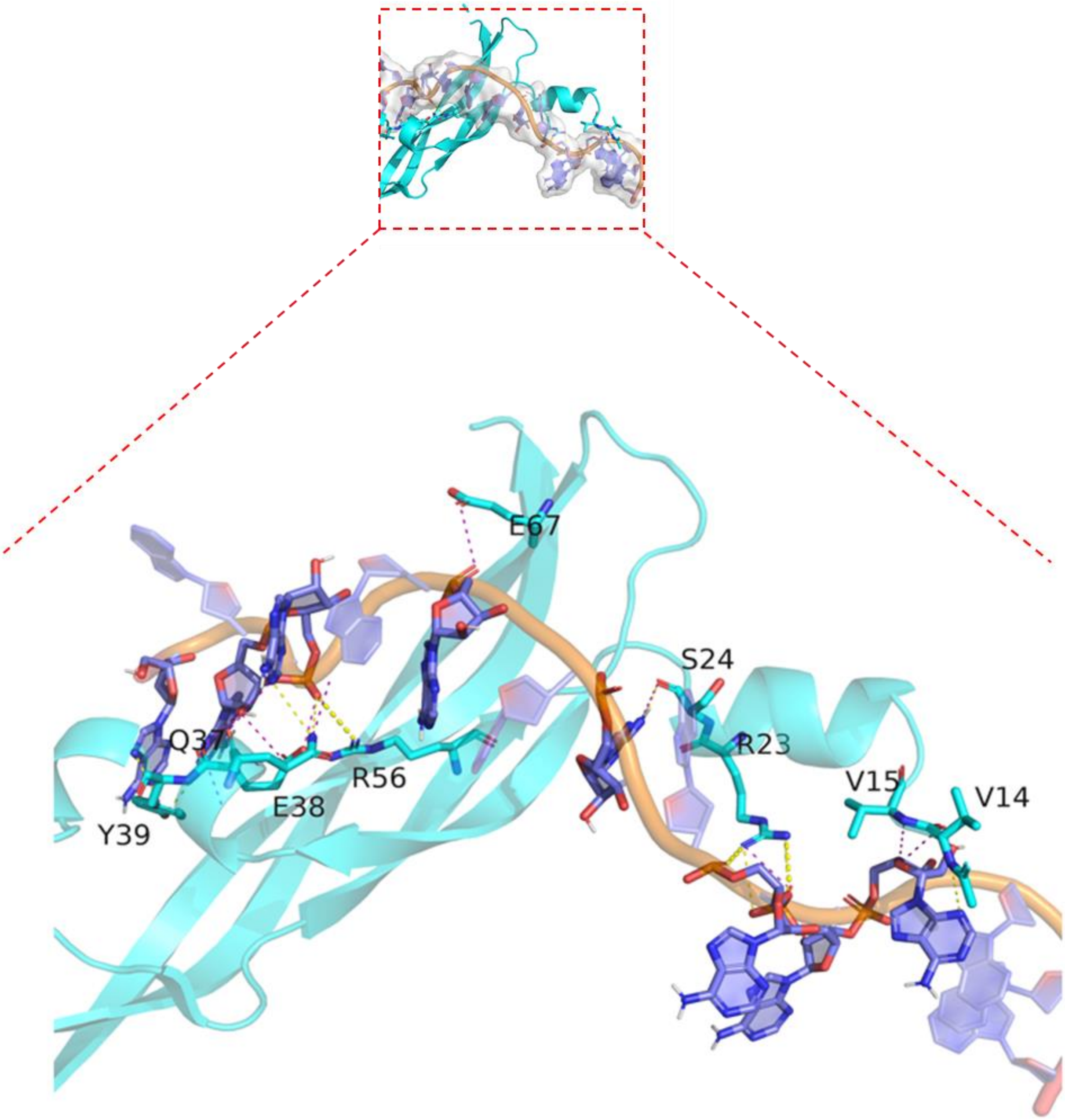

Figure S8. Binding pattern between RNA$_{(15nt)}$ and VEGF. Hydrogen bonds are shown as yellow dotted lines, and van der Waals contacts within 4 Å are shown as magenta dotted lines. The O atom is red, the N atom is blue, the H atom is white, and the S atom is yellow. Protein uses ribbon display mode and RNA uses transparent surface display mode and stick display mode. PyMOL software is used to draw pictures and interact.

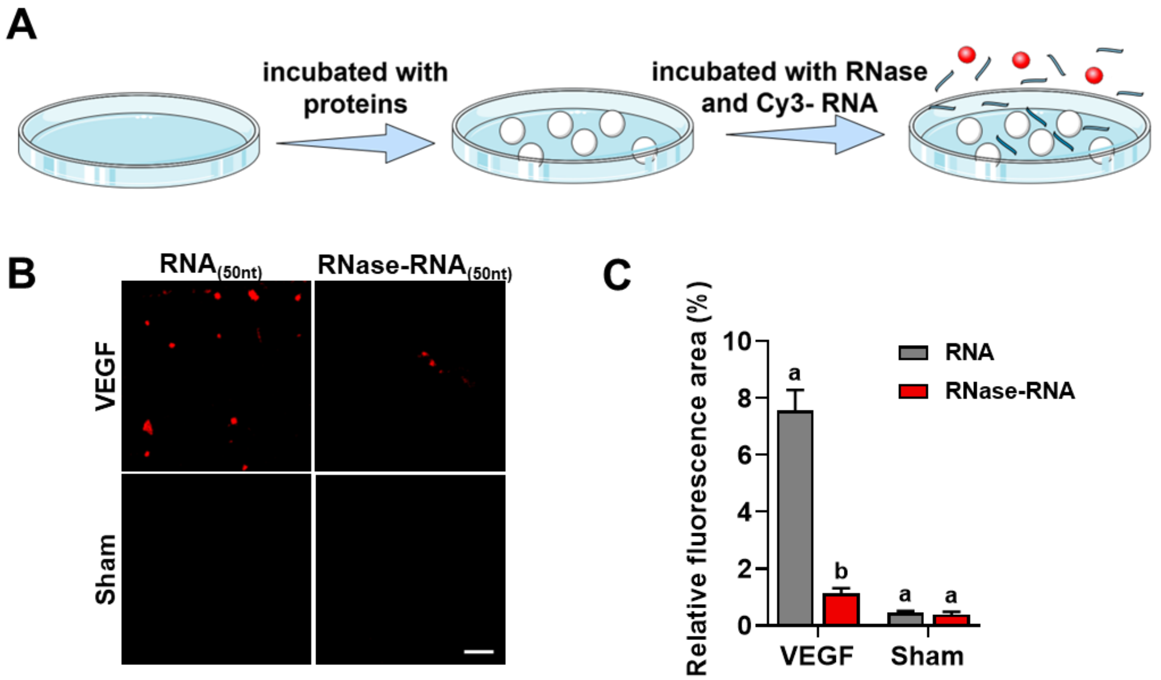

Figure S9. RNase disturbs RNA$_{(50nt)}$-VEGF complex. A Schematic depicting the design and evaluation of the *in vitro* experiments. **B** CLSM images indicate RNase disturbs the combination between RNA$_{(50nt)}$ and VEGF. Scale bars = 30 μm. **C** The data in (**B**) were analyzed quantitatively. Data are shown as the means and standard deviations; $p < 0.05$ ($n = 3$). Equivalent means have the same letter; different letters indicate statistically significant differences.

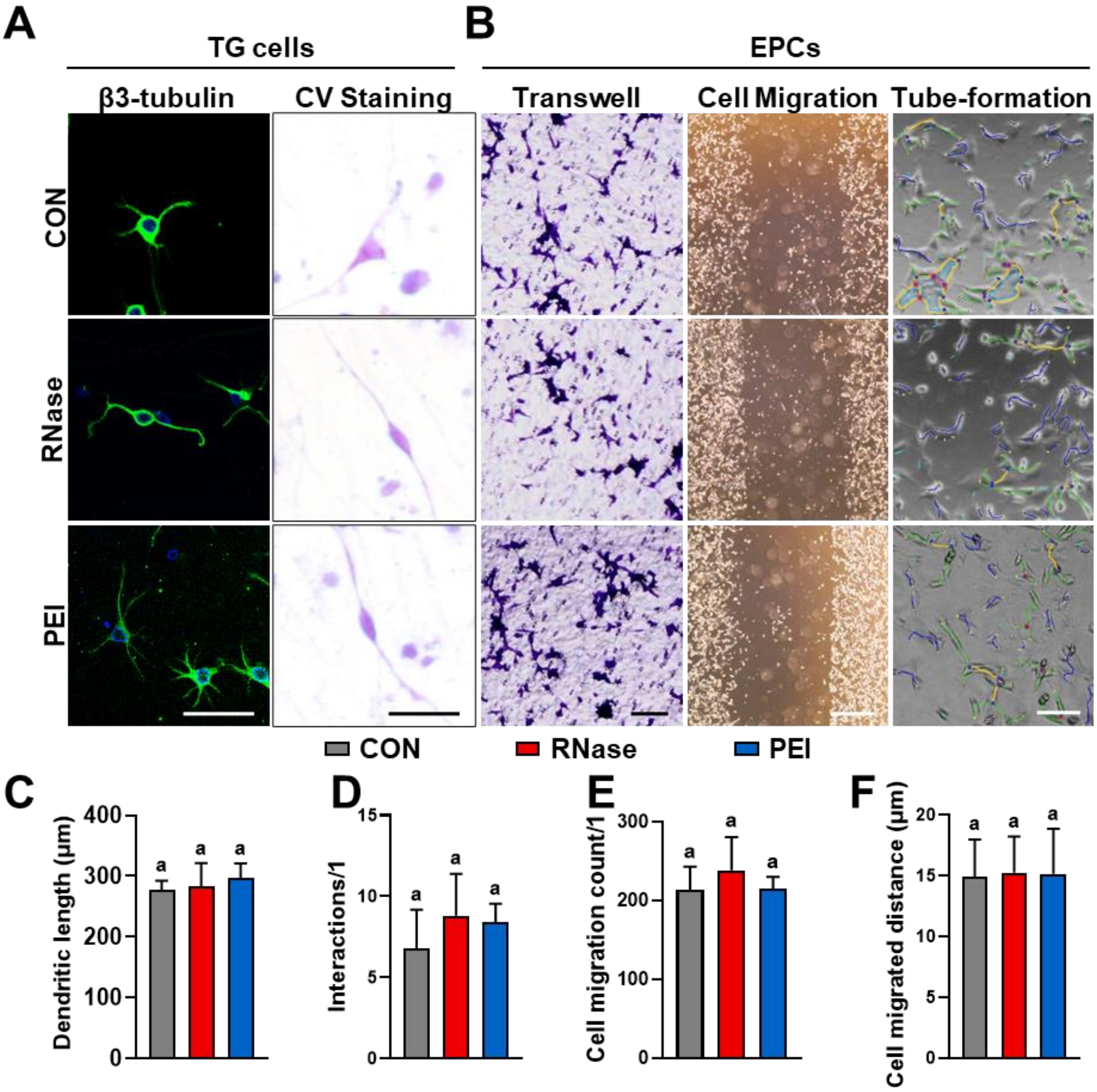

Figure S10. RNase or PEI along doesn't have inhibitory effect on the function of TG cells and EPCs. **A** Representative microscopy images of TG cells show the dendritic length and interactions after 24 h treatment. **B** Representative light microscopy images of EPCs show migrated cells, cell migration and tube formation after 24 h treatment. **C-D** The quantification of dendritic length and interactions of TGs. **E-F** The quantification of the numbers and distance of migrated EPCs. Scale bars = 50 μm (**A**), 100 μm (left and right in **B**), and 500 μm (middle in **B**). Data are shown as the means and standard deviations; $p < 0.05$ (n = 3). Equivalent means have the same letter; different letters indicate statistically significant differences.

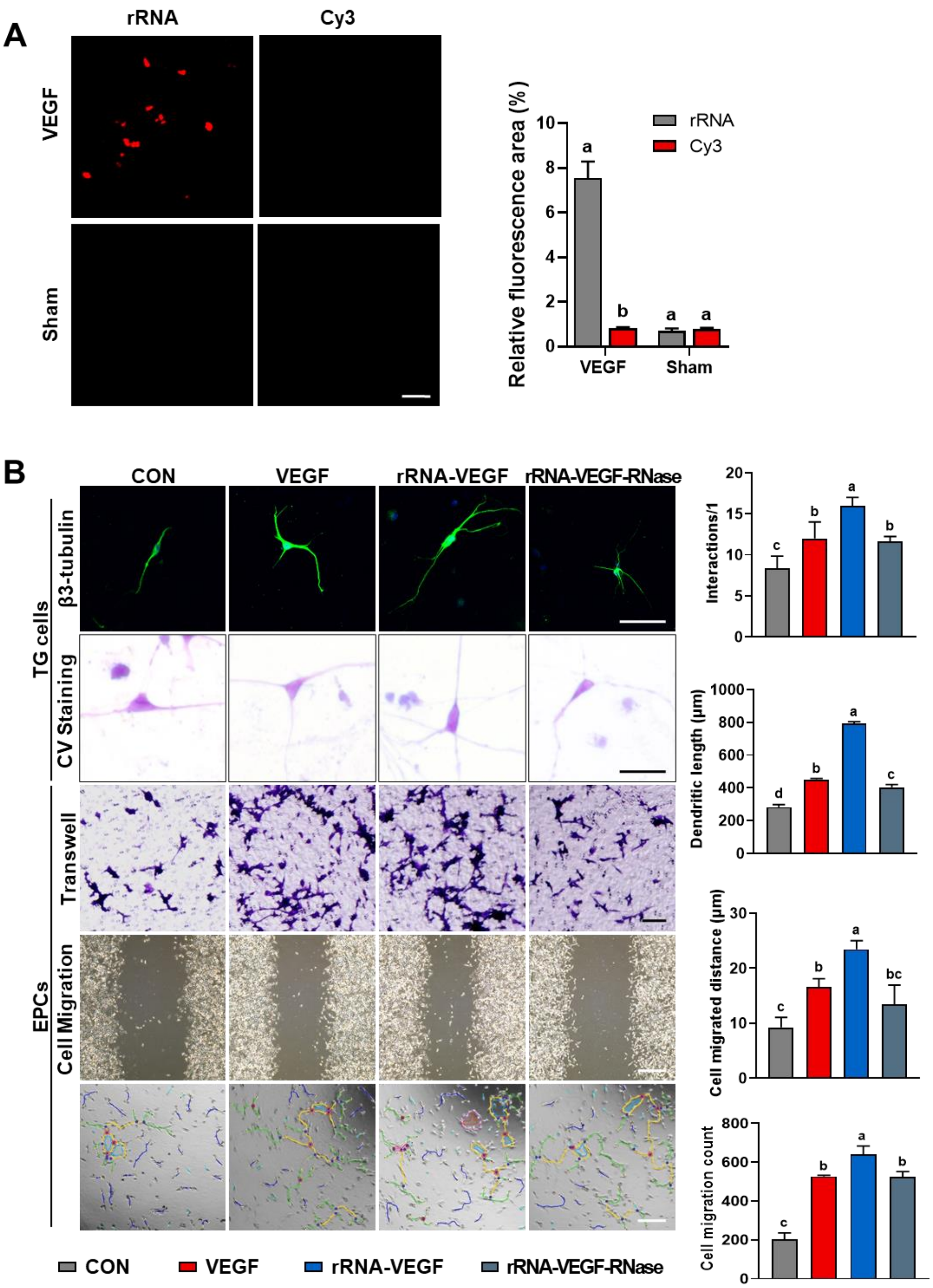

Figure S11. rRNA have the similar effect on the function of VEGF. A CLSM images and statistical analyses indicate rRNA can bind with VEGF. B Representative microscopy images of TG cells and EPCs. Scale bars = 30 μm (**A**), 100 μm (top and bottom in **B**), and 500 μm (middle in **B**). Data are shown as the means and standard deviations; $p < 0.05$ ($n = 3$). Equivalent means have the same letter; different letters indicate statistically significant differences.

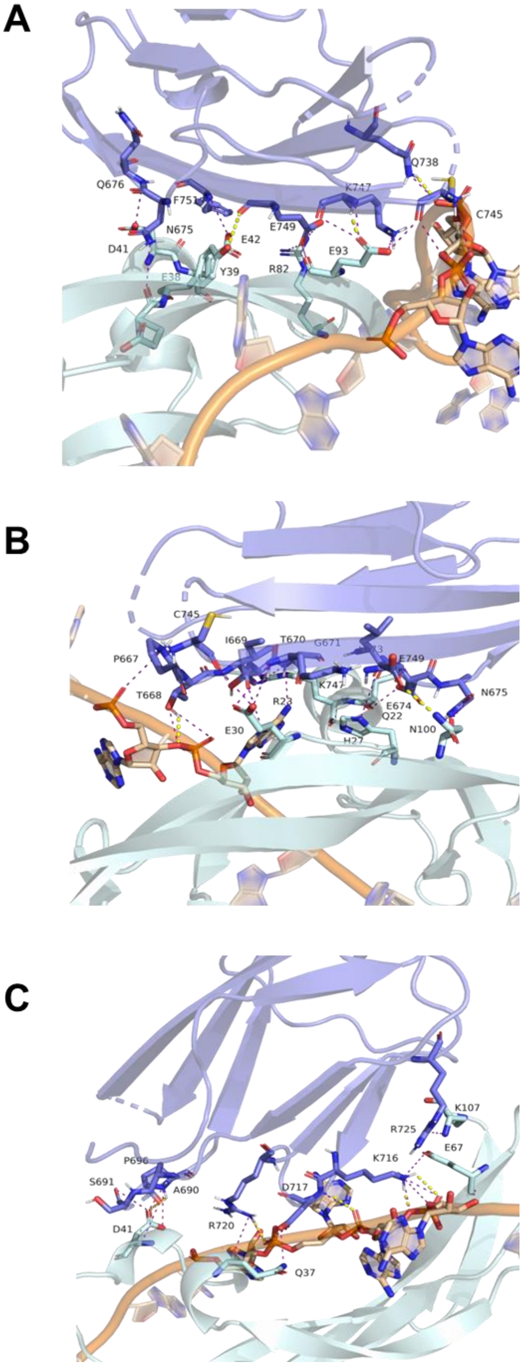

Figure S12. Binding pattern between RNA$_{(50nt)}$-VEGF and VEGFR2 extracellular segment. A, B, and C represent Site1, Site2, and Site3 respectively. VEGFR2 extracellular segment is displayed in blue ribbon, VEGF is displayed in cyan ribbon, and RNA is displayed in orange ribbon. Hydrogen bonds are shown as yellow dotted lines, and van der Waals contacts within 4 Å are shown as purple dotted lines. The O atom is red, the N atom is blue, the H atom is white, and the S atom is yellow, the P atom is dark yellow.

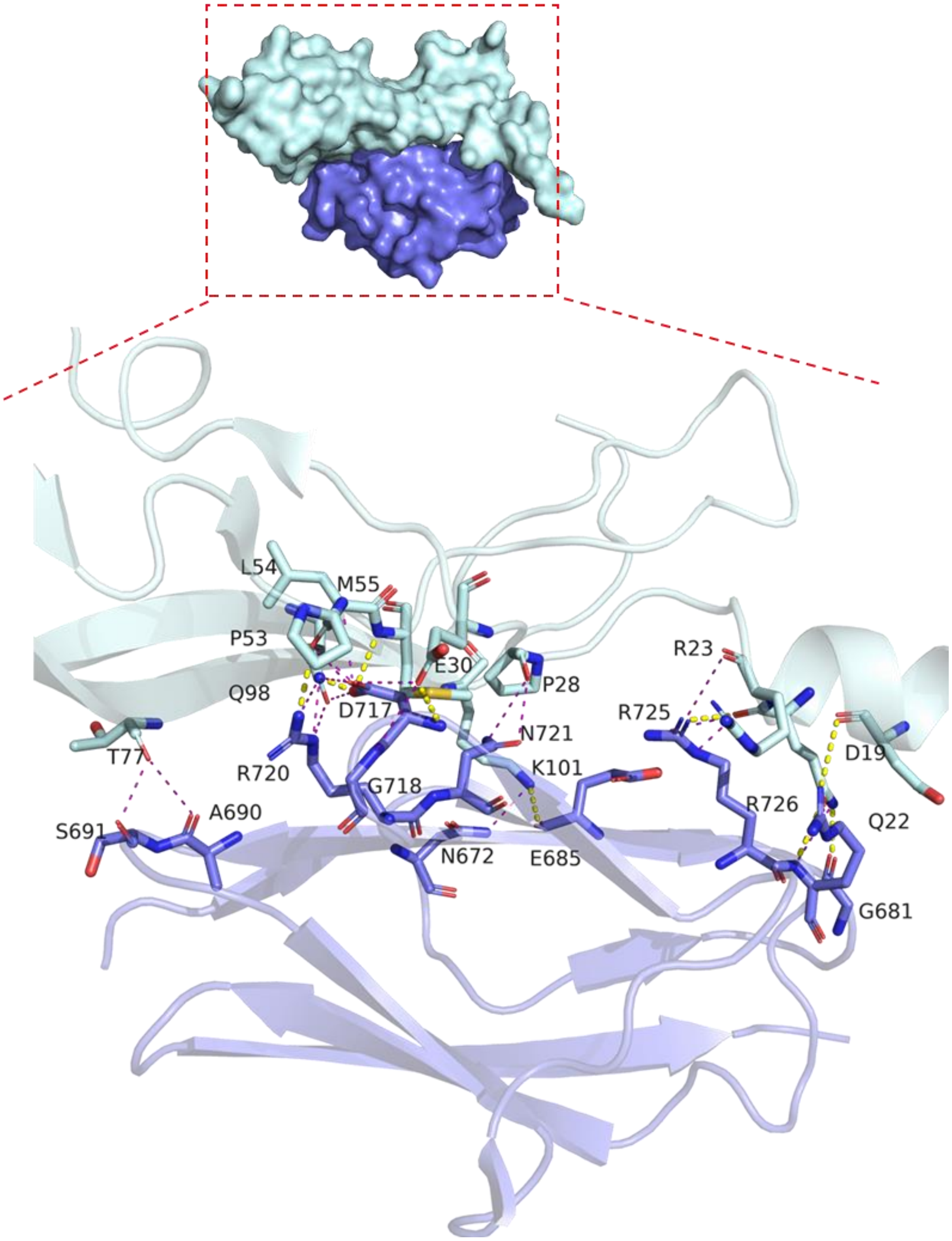

Figure S13. Binding pattern of VEGF and VEGFR2 extracellular segment. VEGFR2 extracellular segment is displayed in blue ribbon, and VEGF is displayed in cyan ribbon. Hydrogen bonds are shown as yellow dotted lines, and van der Waals contacts within 4 Å are shown as purple dotted lines. The O atom is red, the N atom is blue, the H atom is white, and the S atom is yellow, the P atom is dark yellow.

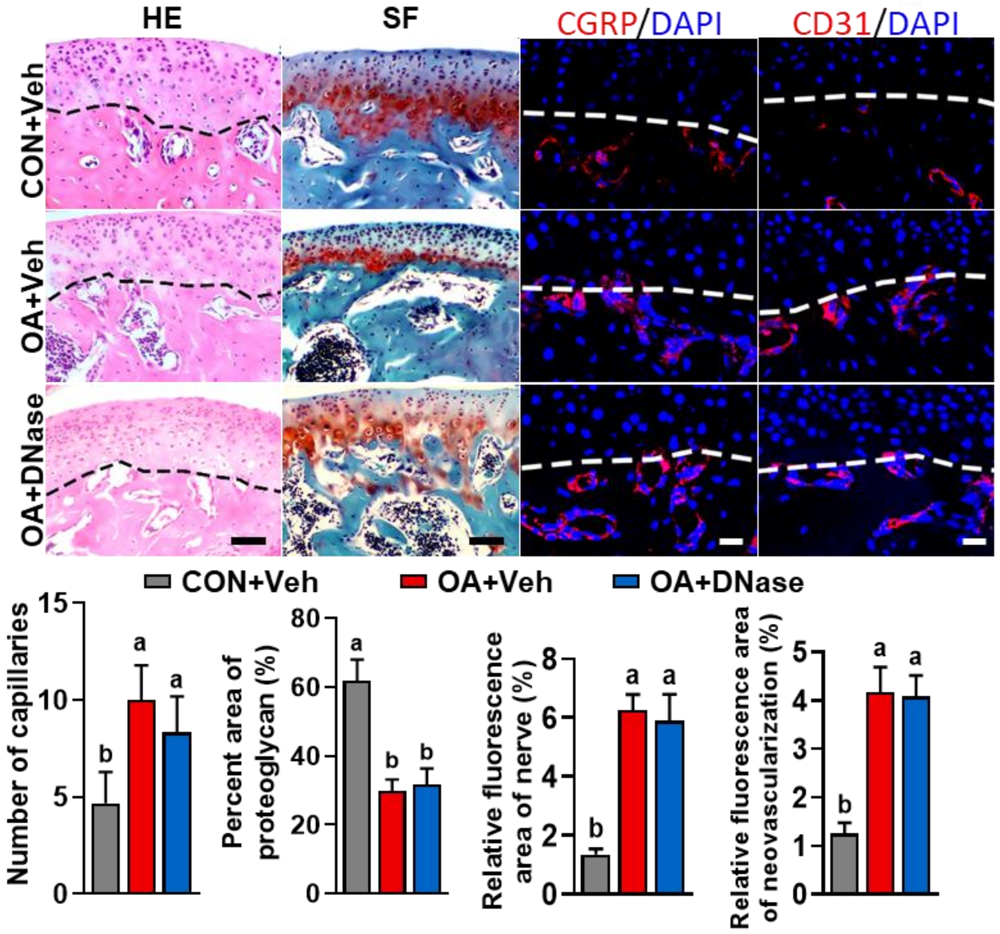

Figure S14. DNase doesn't reverse the progression of OA. Representative images of HE staining, SF staining, nerves (CGRP, red) and vessels (CD31, red) of the mice condyles in different groups at 3 weeks and its statistical analysis. Veh refers to vehicle (i.e., saline). Scale bars =70 μm (HE and SF), 20 μm (IF). Data are shown as the means and standard deviations; $p < 0.05$ ($n = 3$). Equivalent means have the same letter; different letters indicate statistically significant differences.

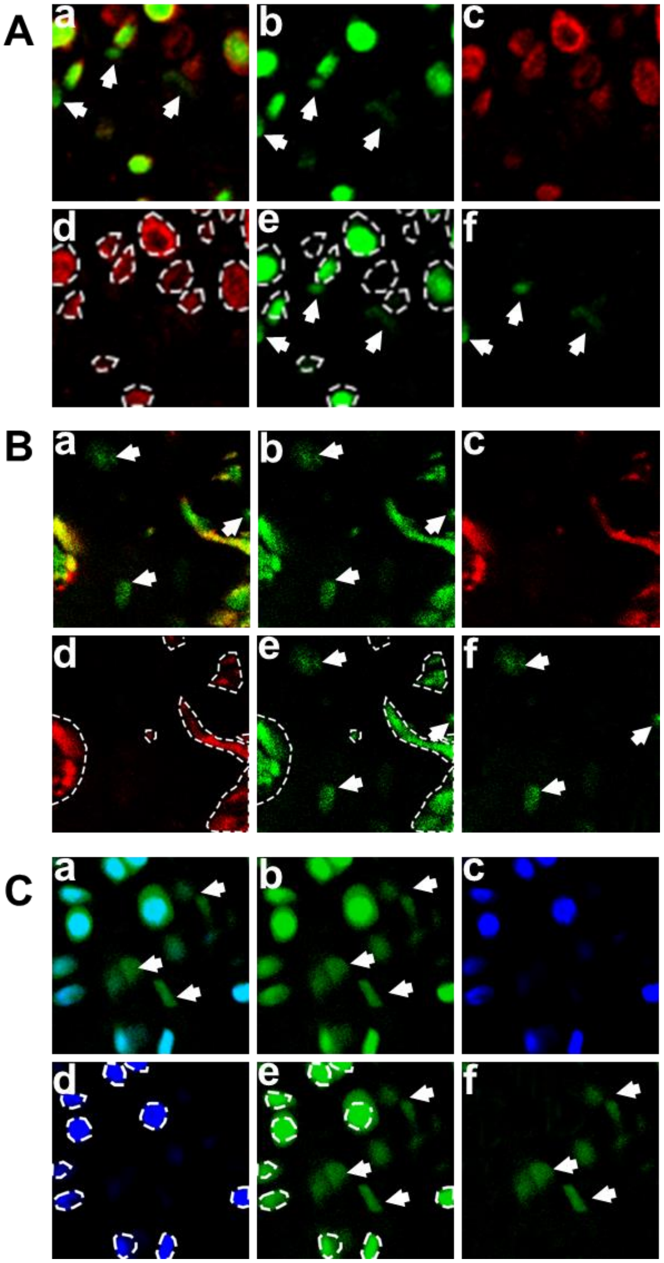

Figure S15. The steps of exRNA quantification in Figure 2 sections. Sections are stained with SYTO® RNASelect™ Green Fluorescent Cell Stain (a RNA-specific stain, green) and co-stained with cytomembrane (E-cadherin, red, A), cytoskeleton (α-tubulin, red, B) or nucleus (DAPI, blue, C) markers. (**a**) Sections displayed in Figure 2. (**b**) RNA channel from **a**. (**c**) E-cadherin channel (**A**), α-tubulin channel (**A**) and DAPI channel (**A**) from **a**. (**d**) Dotted lines mark the fluorescent area in **c**. (**e**) Dotted lines are merged with RNA channel. Then, the RNA around dotted lines is subtracted from **e** to yield the image shown in panel **f** that shows the fluorescence area of "exRNA".

Table S2. Calculation of binding free energy between VEGF protein and RNA (kcal/mol).

	VEGF-RNA$_{(50nt)}$-Site 1	VEGF-RNA$_{(50nt)}$-Site 2	VEGF-RNA$_{(50nt)}$-Site 3	VEGF-RNA$_{(15nt)}$
ΔE_{vdw}	-83.59±4.97	-82.70±6.07	-82.89±5.38	-81.55±5.93
ΔE_{elec}	-291.89±21.08	-285.13±17.67	-280.29±36.65	-280.58±27.38
ΔE_{GB}	210.13±19.21	210.29±14.93	213.42±36.72	213.13±27.70
ΔE_{surf}	-10.35±0.59	-9.1030±0.5534	-8.4387±0.366	-10.7189±0.4356
ΔG_{GAS}	-375.48±20.84	-357.83±15.42	-363.18±39.80	-362.12±30.55
ΔG_{solv}	199.77±19.28	201.19±15.07	204.98±36.57	202.42±27.32
ΔG_{bind}	-175.71±0.82	-166.64±3.72	-158.20±6.85	-159.71±6.95

Table S3. Primer sequences used for RT-PCR in the present study.

**Gene name**	**Sequence （5’-3’）**
m-Pdgfb-F	ATGAAATGCTGAGCGACCACT
m-Pdgfb-R	TCAGCCCCATCTTCATCTACGG
m-Ngf-F	ACCACGACTCACACCTTTGTCAAG
m-Ngf-R	CACACACACACAGGCCGTATCTATC
m-Vegf-F	ACATTGGCTCACTTCCAGAAACAC
m-Vegf-R	TGGTTGGAACCGGCATCTTTA
m-Hif1α-F	TGGACTTGTCTCTTTCTCCGC
m-Hif1α-R	GACGTTCAGAACTCATCCTATTTT
m-Hif2α-F	GAGGAAGGAGAAATCCCGTGA
m- Hif2α-R	CTGATGGCCAGGCGCATGATG
m-Netrin1-F	GTTCGGCGACGAGAACGAA
m-Netrin1-R	TGTGCCTACAGTCACACACCAGA
m-Netrin3-F	AGGTCGGCCAATCGCGT
m-Netrin3-R	CAGGGGCACTGTGAGGGTTAC
m-Netrin4-F	AGATCACCAACCTCCGAGTGC
m-Netrin4-R	GCCCTTGACGATGAAGTCATAGAC
m-Slit1-F	AGGGCCATGTCCGTGTTAGTTA
m-Slit1-R	CGCTGTTGAGGGTGTAGTGCTT
m-Slit2-F	GGCGGTGTCCTCTGTGATGA
m-Slit2-R	CTGCTGCTTCTGGTAATAGTCCCT
m-Slit3-F	AGACCCTGAACCTGGTGGTAGAC
m-Slit3-R	TCTGCACCCTGGCGTAAGG
